# Amidinate- and Dithiolene-Based Silicon Complexes

**DOI:** 10.1021/acs.organomet.5c00038

**Published:** 2025-03-19

**Authors:** Yuzhong Wang, John C. Johnson, Kayla G. Palmer, Pingrong Wei, Earle R. Adams, Mitchell E. Lahm, Henry F. Schaefer, Gregory H. Robinson

**Affiliations:** Department of Chemistry and Center for Computational Chemistry, The University of Georgia, Athens, Georgia 30602-2556, United States

## Abstract

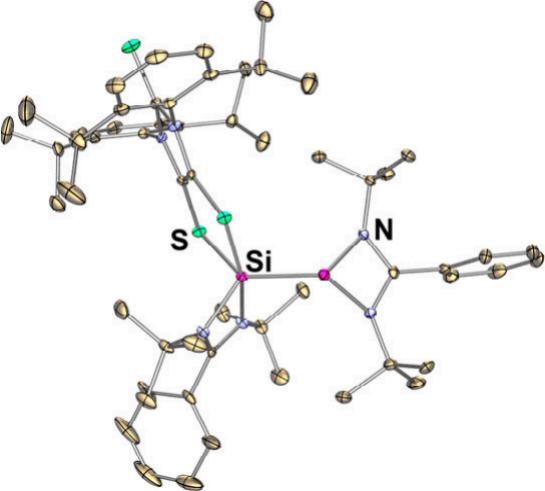

Reactions of the amidinato-silylene chloride PhC(^*t*^BuN)_2_SiCl (**1**) with imidazole-based dithione dimer **2**, lithium dithiolene radical **3**, and dithiolate dimer **4** result in the synthesis of a series of silicon complexes **5**–**7**, respectively, containing both amidinato and dithiolene ligands. **7** is the first structurally characterized silicon(II) dithiolene complex. The structural and bonding characteristics of **5**–**7** have been probed by both experimental and theoretical methods.

## Introduction

**Figure 1:**
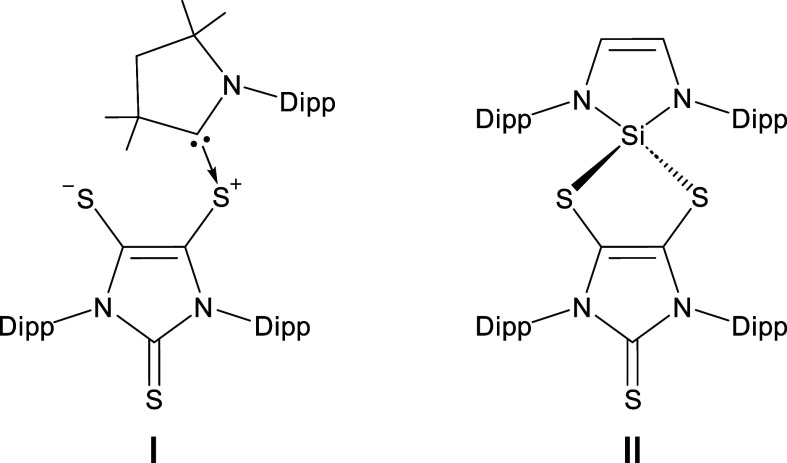
Lewis base–dithiolene complexes (Dipp = 2,6-diisopropylphenyl): (**I**) carbene-stabilized dithiolene zwitterion; (**II**) spirocyclic dithiolene-based N-heterocyclic silane.^3^

Dithiolenes are well-documented as redox-noninnocent ligands in transition metal complexes.^1,2^ This laboratory is exploring the chemistry of transition-metal-free dithiolene molecular systems,^3,4^ wherein the cyclic(alkyl)(amino)carbene (CAAC)-stabilized dithiolene zwitterion **I** (Figure 1) has proven intriguing.^3^ For example, zwitterion **I** has demonstrated a unique utility in ammonia activation via single electron transfer (SET) and hydrogen atom transfer (HAT).^5^ In addition, **I** has been shown to mediate B–H bond activation of BH_3_·SMe_2_ via hydride-coupled reverse electron transfer (HCRET).^6^

N-heterocyclic silylene (NHSi)^7^ has demonstrated different reactivity toward the neutral dithiolene species than carbenes, affording the spirocycle (**II**) (Figure 1).^3^ Further reactivity studies of **II** with boron halides reveal that the dithiolene-capped NHSi possesses an unusual nucleophilic backbone carbon.^8^ In an effort to extend the emerging chemistry at the silylene–dithiolene interface, herein we report the syntheses,^9^ structures,^9^ and computations^9^ of amidinate- and dithiolene-based silicon complexes (**5**–**7**). While previously reported mono-, bis-, and tris(dithiolene) complexes predominantly contain a silicon(IV) core,^3,8,10−12^**7** is the first structurally characterized silicon(II) dithiolene complex.

## Results and Discussion

**Figure 2:**
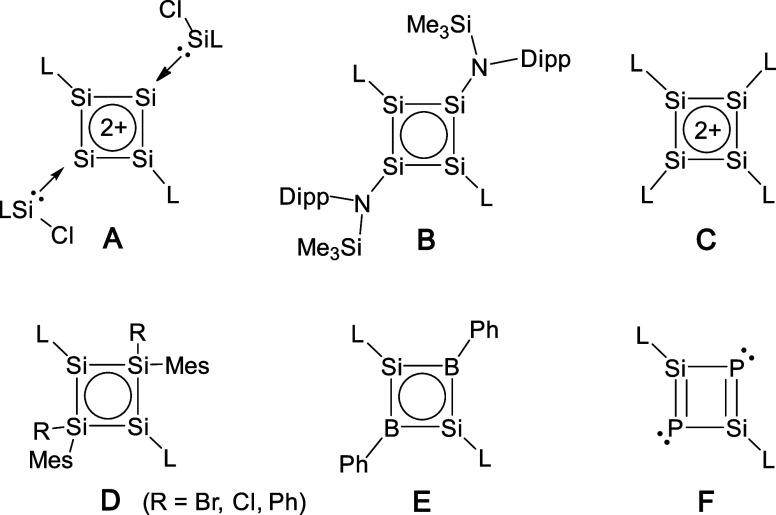
Amidinato-silylene-based low-oxidation-state main group clusters **A**–**F** (L = PhC(^*t*^BuN)_2_; Dipp = 2,6-diisopropylphenyl).

**Scheme 1:**
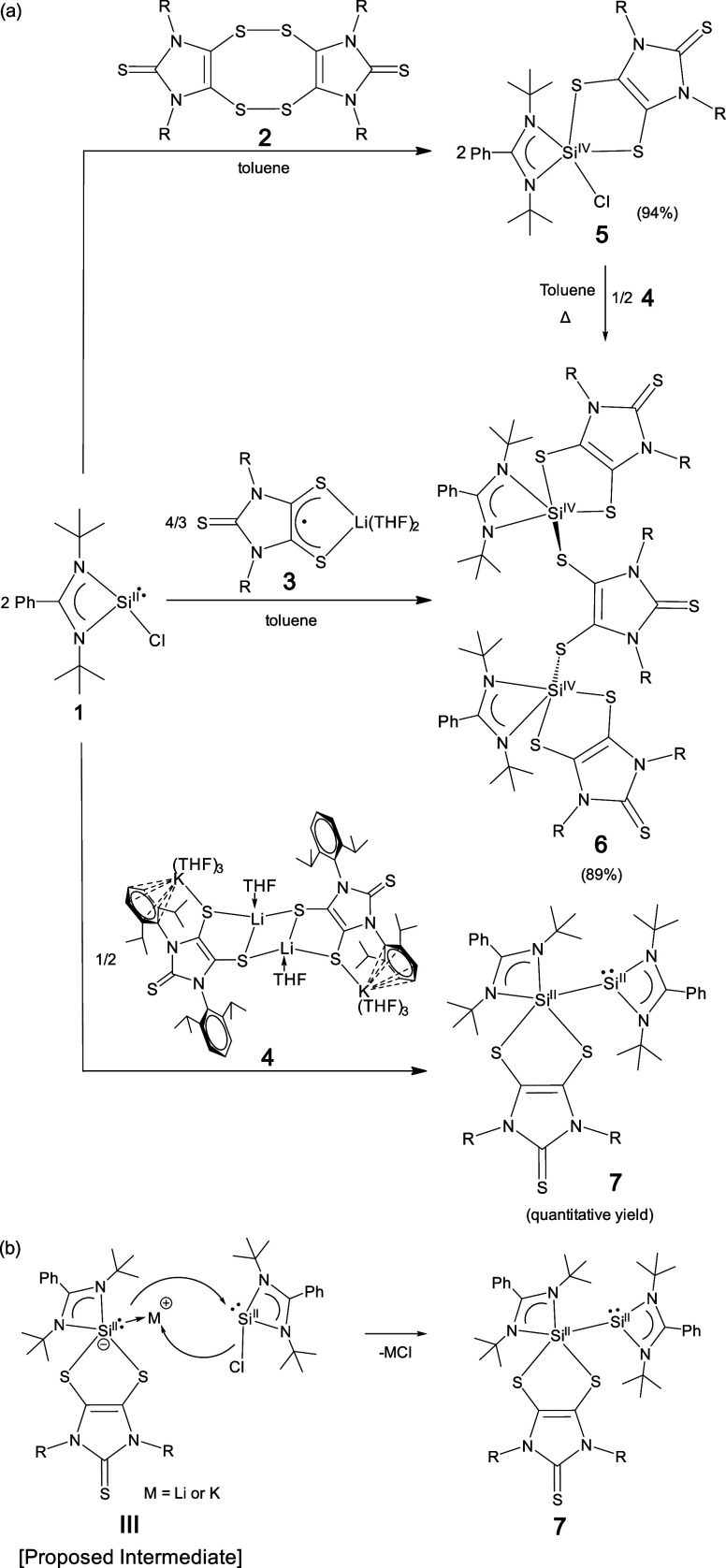
(a) Synthesis of **5**–**7** (R = 2,6-Diisopropylphenyl); (b) Proposed Mechanism for the Formation of **7**

The amidinato-silylene chloride PhC(^*t*^BuN)_2_SiCl (**1**)^13,14^ (Scheme 1a) has exhibited unique utility in the synthesis of bissilylenes^15−21^ and in accessing various low-oxidation-state main group clusters (such as **A**–**F** in Figure 2).^16,22−32^ However, the synthetic applications of **1** have not been extended to dithiolene-based main group chemistry. This laboratory recently reported a series of imidazole-based dithiolene ligands at different redox levels: dithione (L^0^) dimer **2**,^33^ lithium dithiolene radical (L^•–^) **3**,^34^ and dithiolate dimer (L^2–^) **4** (L = dithiolene ligand).^33^ Exploring the reactivity of **1** as a function of dithiolenes **2**–**4** is a logical extension of this research.

Reaction of **1** with dithione dimer **2** (in a 2:1 molar ratio) in toluene gives **5** as a colorless crystalline solid in 94% yield (Scheme 1a).^9^ X-ray-quality colorless crystals of **5** were obtained by recrystallization in toluene. The ^29^Si NMR resonance of **5** (−69.5 ppm, in THF-*d*_8_) shows a downfield shift compared to that of dioxolane-complexed **1** (−92.2 ppm, in C_6_D_6_).^35^ Further reaction of **5** with **4** (in a 4:1 molar ratio) in toluene at an elevated temperature gives **6** in 89% yield (Scheme 1a). Compound **6** may also be directly prepared (in lower yield) by the reaction of **1** and dithiolene radical **3** (in a 3:2 molar ratio) (Scheme 1a). Slow diffusion of hexane into a concentrated THF solution of **6** at room temperature yielded colorless X-ray-quality crystals. The ^29^Si NMR resonance of **6** (−59.0 ppm, in PhBr-*d*_5_) approaches that of **5** (−69.5 ppm), supporting the presence of the five-coordinate silicon atom in **6**.^36^

The 4:1 reaction of **1** with dithiolate dimer **4** in THF gives **7** in quantitative yield (Scheme 1a). X-ray-quality yellow crystals of **7** were obtained from a concentrated toluene solution. The ^29^Si NMR spectrum of **7** (in THF-*d*_8_) exhibits two resonances at +17.6 and −51.7 ppm, which correspond to the three-coordinate^13,14^ and five-coordinate^36^ silicon atoms, respectively. Compounds **6** and **7** are extremely air- and moisture-sensitive. The synthesis of **7** may involve the formation of the anionic silylene intermediate **III** (Scheme 1b) first via a salt elimination reaction between 1 equiv of **1** and 0.5 equiv of **4**. Subsequently, **III** reacts with another 1 equiv of **1** via a second salt elimination to give **7**. Attempts to obtain the proposed intermediate **III** were unsuccessful. The NMR tube reactions show that the parallel 2:1 reaction of **1** and dimer **4** (in THF-*d*_8_) gives a mixture of **7** and unreacted **4**. This result suggests that the anionic silylene center in intermediate **III** is a stronger nucleophile than dithiolates.

**Figure 3:**
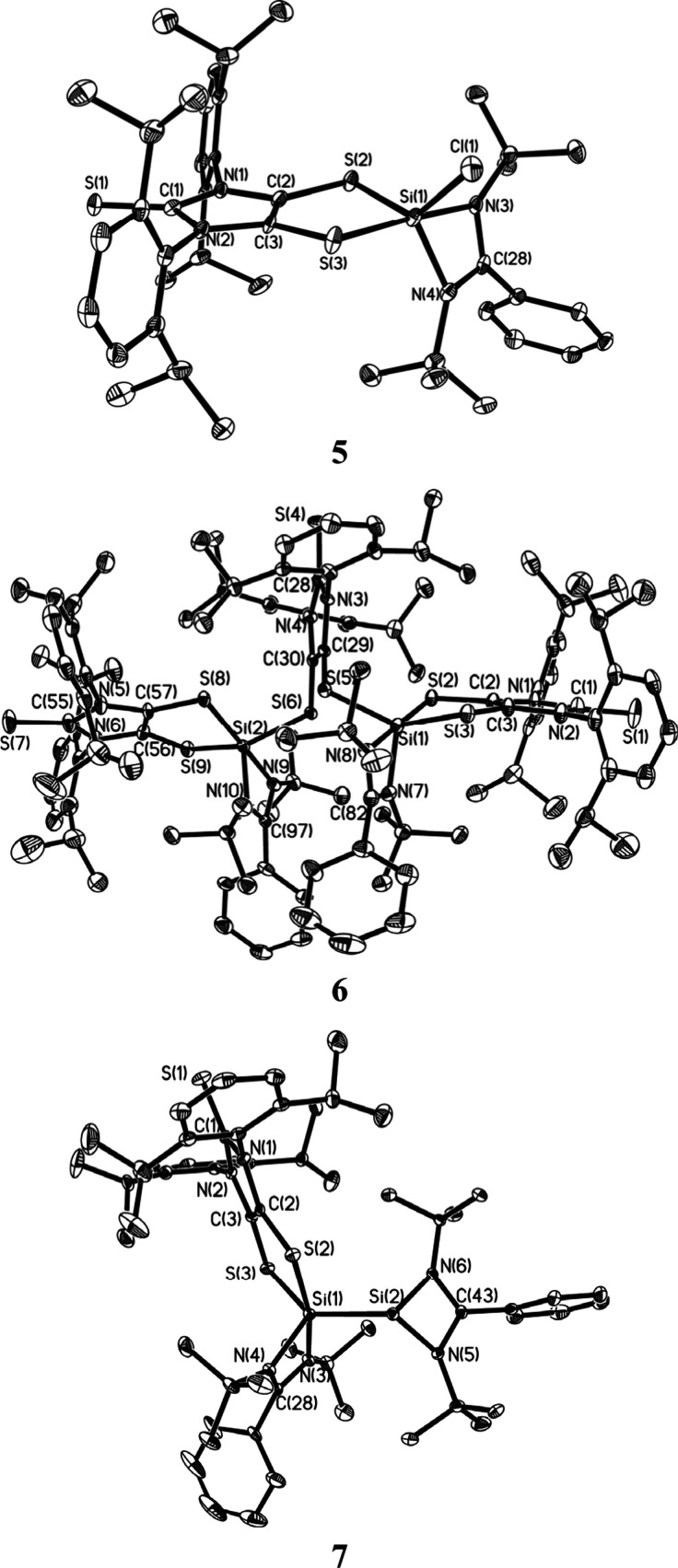
Molecular structures of **5**, **6**, and **7**. Thermal ellipsoids represent 30% probability. Hydrogen atoms have been omitted for clarity. Selected bond distances (Å) and angles (deg) for **5**: C(2)–C(3) 1.333(6); C(2)–S(2) 1.722(5); Si(1)–S(2) 2.1717(18); Si(1)–S(3) 2.2498(18); Si(1)–N(3) 1.906(4); Si(1)–N(4) 1.805(4); Si(1)–Cl(1) 2.0921(19); Cl(1)–Si(1)–S(2) 130.90(8); N(3)–Si(1)–S(3) 176.23(15). Selected bond distances (Å) and angles (deg) for **6**: C(2)–C(3) 1.339(8); C(2)–S(2) 1.739(6); Si(1)–S(2) 2.258(2); Si(1)–S(3) 2.200(2); Si(1)–S(5) 2.175(2); Si(1)–N(7) 1.823(5); Si(1)–N(8) 1.933(5); C(29)–C(30) 1.343(7); C(29)–S(5) 1.740(6); N(8)–Si(1)–S(2) 178.88(17); S(3)–Si(1)–S(5) 137.07(10). Selected bond distances (Å) and angles (deg) for **7**: C(2)–C(3) 1.346(5); C(2)–S(2) 1.728(3); Si(1)–S(2) 2.4291(13); Si(1)–S(3) 2.1987(13); Si(1)–N(3) 1.952(3); Si(1)–N(4) 1.838(3); Si(1)–Si(2) 2.3966(14); Si(2)–N(5) 1.870(3); Si(2)–N(6) 1.875(3); S(2)–Si(1)–N(3) 169.20(10); Si(2)–Si(1)–S(3) 138.11(5).

The molecular structure of **5** (Figure 3)^9^ confirms **1**-mediated cleavage of the sulfur–sulfur bonds in **2**, giving both amidinate- and dithiolene-complexed silicon chloride (SiCl). The five-coordinate silicon atom in **5** adopts a distorted trigonal-bipyramidal geometry (τ = 0.76).^37^ The C=C bond [1.333(6) Å] and C–S bonds (1.724 Å, av) in the C_2_S_2_ unit of **5** compare well to those for the reported dithiolates (L^2–^).^12,38^ The five-membered C_2_S_2_Si ring in **5** is slightly puckered (the bend angle (η) between the SiS_2_ plane and the C_2_S_2_ plane is 9.8°). The Si–S_ax_ [i.e., S(3)] distance [2.2498(18) Å] and Si–N_ax_ [i.e., N(3)] distance [1.906(4) Å] are longer than the Si–S_eq_ [i.e., S(2)] distance [2.1717(18) Å] and Si–N_eq_ [i.e., N(4)] distance [1.805(4) Å], respectively. The Si–Cl bond [2.0921(19) Å] in **5** is comparable with that in the dioxolane-complexed **1** analogue [2.0958(7) Å].^35^ NBO natural population analysis reveals a positive charge of +1.37 for the silicon atom in **5**. The Si–S_ax_ bond polarization (30.3% toward Si and 69.7% toward S) is slightly larger than that of the Si–S_eq_ bond (32.1% toward Si and 67.9% toward S).^9^

Compound **6** exists as a pair of enantiomers (with identical bonding parameters);^9^ however (for clarity), only one enantiomer of **6** (with its selected bonding parameters) is presented in Figure 3. The existence of enantiomers may be ascribed to the steric congestion of **6**, which restricts the rotation about the two C–S bonds of the C_2_S_2_ unit in the bridging dithiolene ligand. While each is coordinated by one amidinate ligand and one dithiolene ligand, the two silicon atoms are anchored on opposite sides of the imidazole plane of the bridging dithiolene ligand via the Si–S bonds. The S(5)–C(29)–C(30)–S(6) torsion angle in **6** (21.3°) is much larger than that of the simplified **6-H** model [the S–C–C–S torsion angle of the bridging dithiolene ligand = 10.4°],^9^ which may be largely due to the steric repulsion between the bulky ligands. The structural parameters of the amidinate- and dithiolene-complexed silicon units in **6** compare well to those in **5**. Each five-coordinate silicon atom in **6-H** bears an NBO positive charge of +1.29.^9^

**Figure 4:**
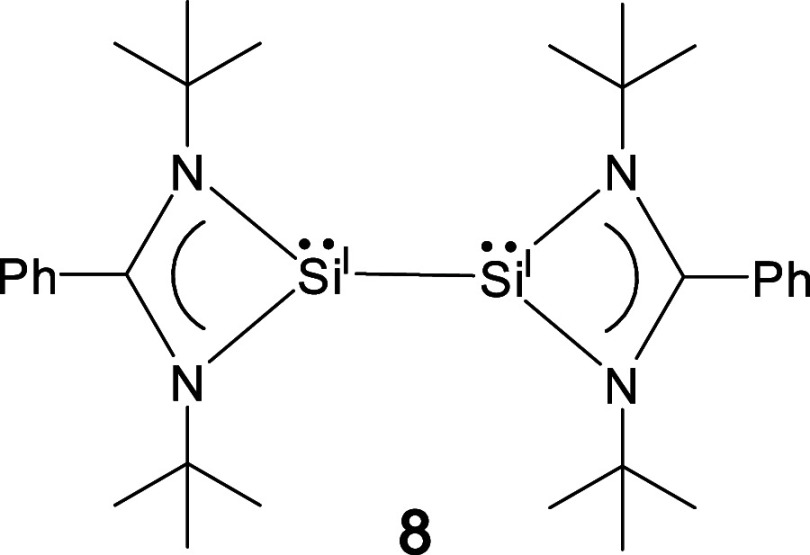
Amidinato-based bis(silylene) **8**.

The unit cell of the crystals of **7** contains an enantiomeric pair (with identical bonding parameters).^9^ For clarity, only one enantiomer of **7** (with its selected bonding parameters) is shown in Figure 3. The presence of enantiomeric **7** may be due to the steric bulk of the ligands, which restricts the rotation about the Si–Si bond. Roesky and co-workers reported an amidinato-bis(silylene) with a Si^I^–Si^I^ bond (**8**) (Figure 4).^39^ Compound **7** may be regarded as a monodithiolene-complexed **8**. Consequently, **7** contains both three-coordinate and five-coordinate silicon atoms.^9^ The geometry of the five-coordinate silicon atom in **7** (τ = 0.52) is more distorted from a perfectly trigonal-bipyramidal geometry than those in **5** (τ = 0.76) and **6** (τ = 0.70, av).^37^ The three-coordinate silicon atom in **7** adopts a trigonal-pyramidal geometry. In **7**, the N–Si(2) bonds [1.870(3) and 1.875(3) Å] are in-between the N_ax_–Si(1) bond [1.952(3) Å] and N_eq_–Si(1) bond [1.838(3) Å]. The Si–Si bond in **7** [2.3966(14) Å] is marginally shorter than the Si^I^–Si^I^ bond [2.413(2) Å] of **8**.^39^ The Mayer bond index^40^ (MBI) and Wiberg bond index (WBI) values of the Si–Si bond in **7** are 0.818^41,42^ and 0.897, respectively. The WBI value of the Si–Si bond in **7** (0.897) is somewhat smaller than that for **8** (0.977).^35^ The three- and five-coordinate silicon atoms [i.e., Si(2) and Si(1)] in **7** bear positive charges of +0.72 and +1.03, respectively. The oxidation states of the silicon atoms in **5**, **6-H**, and **7** were evaluated by the localized orbital bonding analysis (LOBA)^43^ method using Multiwfn.^41,42^ While the silicon atoms in **5** and **6-H** are in the +4 oxidation state, both the five-coordinate and three-coordinate silicon atoms in **7** exhibit the +2 oxidation state.

**Figure 5:**
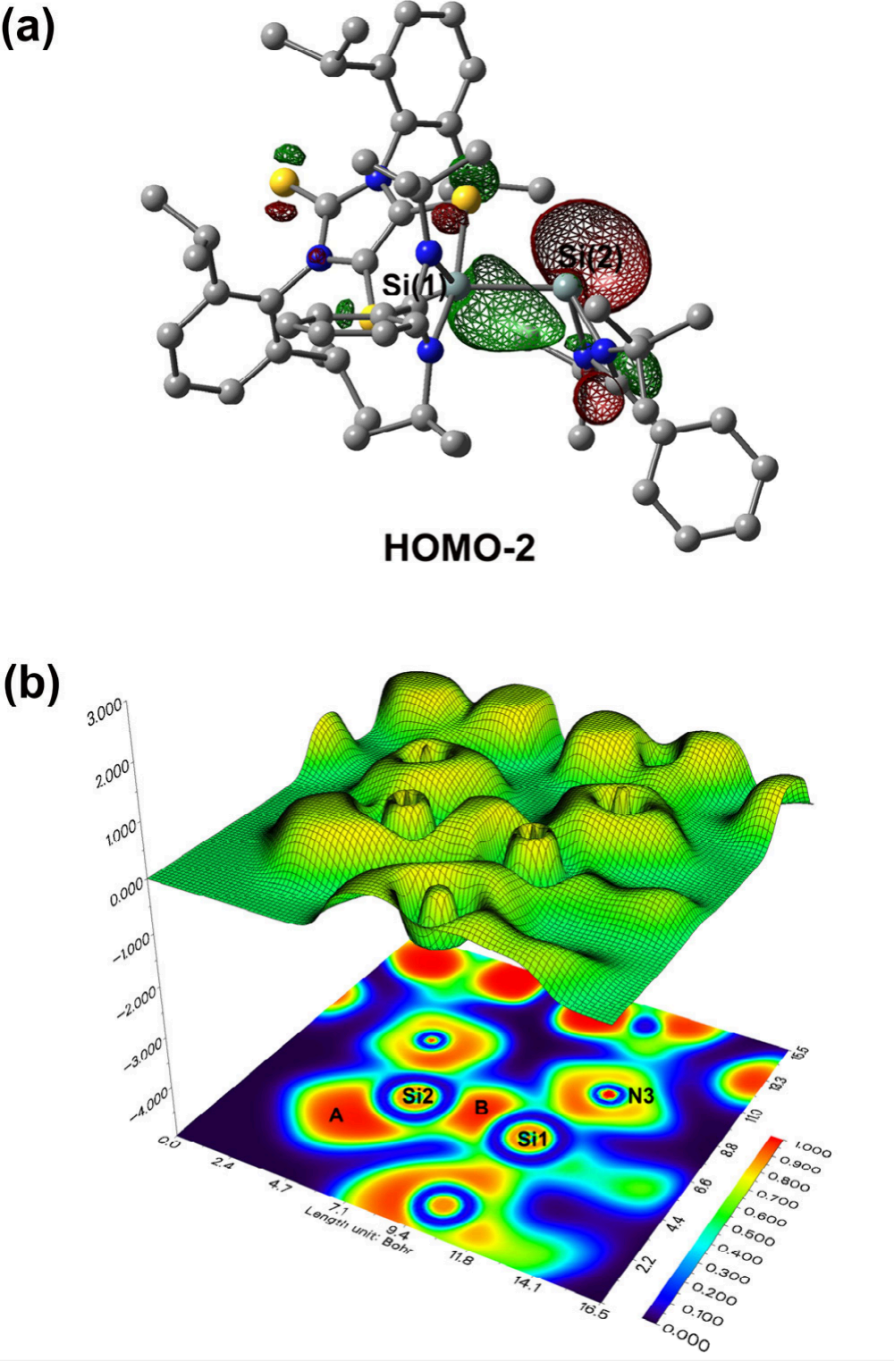
(a) HOMO–2 of **7** (the isosurface value for the orbital plot = 0.04; hydrogen atoms have been omitted for clarity). (b) Shaded relief map with projection effect of the electron localization function of **7** in the Si(2)–Si(1)–N(3) plane.

DFT computations at the B3LYP/6-311G** level reveal that the LUMOs of **5**, **6-H**, and **7** (Figure S10) are mainly involved in the π-antibonding orbital of the phenyl group in the amidinate ligand. However, the HOMOs of **5**, **6-H**, and **7** (Figure S10) are predominantly dithiolene-based, bearing C–C π-bonding and C–S π-antibonding character.^9^ The HOMO–2 of **7** (Figure 5a) involves both Si–Si σ-bonding and Si(2) atom-based lone-pair character. The presence of a Si–Si σ bond (bond region B in Figure 5b) and a Si(2)-based lone pair (lone-pair region A in Figure 5b) in **7** is further supported by the electron localization function (ELF) study using Multiwfn^41,42^ (note: the color scale of the ELF map in Figure 5b varies from blue to red in the range from 0 to 1). According to NBO analysis, the silicon–silicon bond polarization of **7** is 61.4% toward Si(1) and 38.6% toward Si(2). While the five-coordinate Si(1) atom has 44.2% s, 55.5% p, and 0.3% d character, the three-coordinate Si(2) atom owns 25.1% s, 74.3% p, and 0.6% d character.

## Conclusion

Reaction of **1** with dithiolene ligands **2**–**4** affords a series of amidinate- and dithiolene-based silicon(II) and silicon(IV) complexes (**5**–**7**). Compound **7** is the first structurally characterized silicon(II) dithiolene complex. The chemistry of these new compounds is under investigation.

## Supporting Information Available

Syntheses, computations, and X-ray crystal determination (PDF)
Cartesian coordinates for the calculated structures (XYZ)

## Supplementary Material

om5c00038_si_001.pdf

om5c00038_si_002.xyz
