# Supplementary material for: Amidinate- and Dithiolene-Based Silicon Complexes
Source: Organometallics. 2025 Mar 19;44(7):802–6. doi: 10.1021/acs.organomet.5c00038 (PMC12001252; doi:10.1021/acs.organomet.5c00038)
Supplement: Supplementary file 1 — om5c00038_si_001.pdf [file om5c00038_si_001.pdf]

Supplementary Information

Amidinate- and Dithiolene-Based Silicon Complexes

Yuzhong Wang, John C. Johnson, Kayla G. Palmer, Pingrong Wei, Earle R. Adams, Mitchell E. Lahm, Henry F. Schaefer, III, and Gregory H. Robinson*

Department of Chemistry and Center for Computational Chemistry, The University of Georgia; Athens, GA 30602-2556, USA.

*To whom correspondence should be addressed.
Email: robinson@uga.edu

**Table of Contents**	**Page**
Synthesis and spectral data	S2-S12
Computational data	S13-S14
X-Ray Crystallography Data	S15-S30

SUPPORTING INFORMATION of SYNTHESES

Materials and Methods

General.

All reactions were performed under purified argon using Schlenk techniques and an inert atmosphere drybox (M-Braun LabMaster 130). Chemicals were purchased from commercial sources and used as received. Solvents were dried and distilled under argon from Na/benzophenone prior to use. ^{1}H, $^{13}C\{^{1}H\}$, and $^{29}Si\{^{1}H\}$ NMR spectra were recorded on a Bruker Avance III HD 400 MHz spectrometer. The $^{29}Si\{^{1}H\}$ NMR spectra were referenced to TMS as an external standard. X-ray intensity data for **5** (CCDC# 2416719) and **6** (CCDC# 2416720) and **7** (CCDC# 2416721) was collected at 135K on a Bruker D8 Quest PHOTON 100 CMOS X-ray diffractometer system with Incoatec Microfocus Source (IμS) monochromated Mo Kα radiation (λ = 0.71073 Å, sealed tube) using phi and omega-scan technique.

Compounds **5**: 10 mL of toluene was added to a Schlenk tube containing **1** (0.500 g, 1.696 mmol) and **2** (0.818 g, 0.847 mmol). After the mixture were stirred at room temperature overnight, the volatiles were removed in vacuo. The residue was rinsed with 3 mL of hexane and subsequently dried in vacuo, giving **5** as an off-white crystalline solid (1.24 g, 94% yield). X-ray quality colorless single crystals of **5** were obtained by recrystallization in toluene. Characterization of **5**: Mp: 237–240 °C. ^{1}H NMR (400.22 MHz, THF-d_8): δ 1.20 [s, 18H, C(CH_3)$_3$], 1.28–1.30 [m, 24H, CH(CH_3)$_2$], 2.89 [m, 4H, CH(CH$_3$)$_2$], 7.26–7.55 (m, 11H, Ar-H). $^{13}C\{^{1}H\}$ NMR (100.65 MHz, THF-d_8): δ 24.61, [CH(CH$_3$)$_2$], 30.08 [CH(CH$_3$)$_2$], 31.97 [C(CH$_3$)$_3$], 57.26 [C(CH$_3$)$_3$], 120.29 (S–C=C–S), 124.76, 129.18, 129.62, 130.63, 131.90, 132.30, 134.98, 147.99 (Ar-C), 167.62 [NC(Ph)N], 170.66 (C=S). $^{29}Si\{^{1}H\}$ NMR (79.51 MHz, THF-d_8): δ -69.54. Crystal data for **5**: $C_{42}H_{57}N_4S_3SiCl$, fw = 777.63, triclinic, P-1 (No. 2), a = 11.8139(18) Å, b = 13.715(2) Å, c = 14.983(2) Å, α = 80.716(4)°, β = 79.422(4)°, γ = 69.015(4)°, V = 2216.1(6) Å^3, Z = 2, R1 = 0.0946 for 5986 data ($I > 2\sigma(I)$), wR_2 = 0.2429 (all data).

Compound **6**: (*Method 1*) 15 mL of toluene was added to a Schlenk tube containing **4·(THF)** crystals (0.250 g, 0.146 mmol) and **5** (0.456 g, 0.586 mmol). After being stirred at room temperature overnight, the mixture was heated at reflux over one hour. Subsequently, the mixture was filtered at room temperature, giving a yellow filtrate. The volatiles were removed from the filtrate in vacuo, giving raw yellow powder of **6** (0.513 g, 89% yield). X-ray quality single crystals of **6** were obtained from the concentrated parent toluene solution. (*Method 2*) 30 mL of toluene was added to a 100 mL Schlenk flask containing **1** (0.497 g, 1.686 mmol) and **3** (0.713 g, 1.125 mmol). After being stirred at room temperature for 4h, the reaction mixture was filtered and dried under vacuum. The resulting sticky yellow residue was rinsed by 20 mL of hexane, giving a pale-yellow powder. In a Schlenk tube, the pale-yellow solid was dissolved in 5 mL of THF and subsequently covered with 3 mL of hexane, inducing the formation of X-ray quality colorless single crystals of **6** over one week. Mp: gradually decomposed (> 231 °C). ^{1}H NMR (400.22 MHz, PhBr-d_5): δ 0.66–1.62 [m, 108H, C(CH_3)$_3$ and CH(CH_3)$_2$], 2.46 [m, 2H, CH(CH$_3$)$_2$], 2.94–3.05 [m, 6H, CH(CH$_3$)$_2$], 3.23 [m, 2H, CH(CH$_3$)$_2$], 3.41 [m, 2H, CH(CH$_3$)$_2$], 7.20–7.41 (m, 28H, Ar-H). $^{13}C\{^{1}H\}$ NMR (100.65 MHz, PhBr-d_5): δ 22.48, 23.00, 24.26, 24.32, 24.57, 24.76, 24.92, 25.07, 25.39, 25.95, 26.09, 27.31 [CH(CH$_3$)$_2$], 28.46, 28.83, 28.87, 28.91, 29.19 [CH(CH$_3$)$_2$], 29.71, 32.61 [C(CH$_3$)$_3$], 55.76, 56.35 [C(CH$_3$)$_3$], 119.93, 120.17 (S–C=C–S), 123.61, 123.75, 123.94, 125.55, 127.12, 127.67, 127.70, 127.92, 133.24, 133.66, 134.46, 145.63, 146.45, 147.10,

147.35, 148.09, 148.85 (Ar-*C*), 167.29 [N*C*(Ph)N], 167.88 (C=S). ^{29}Si{^{1}H} NMR (79.51 MHz, PhBr-d$_5$): δ -58.95. Crystal data for **6**: $C_{111}H_{148}N_{10}S_9Si_2$, fw = 1967.11, monoclinic, $P2_1/n$ (No. 14), a = 24.3272(18) Å, b = 23.1614(18) Å, c = 27.091(2) Å, β = 105.439(2)°, V = 14714(2) Å^3, Z = 4, R1 = 0.1034 for 15855 data ($I > 2\sigma(I)$), wR$_2$ = 0.2444 (all data).

Compound **7**: 10 mL of THF was added to a Schlenk tube containing **1** (0.316 g, 1.072 mmol) and **4·(THF)** (0.457 g, 0.268 mmol). After the mixture were stirred at room temperature overnight, the volatiles were removed in vacuo, giving raw **7** in a quantitative yield (in terms of the data of the NMR tube reaction). To purify raw **7**, 15 mL of toluene was added to the Schlenk tube. The slurry was then heated at 100 °C for 1h. After being cooled to room temperature, the slurry was filtered. The filtrate was concentrated to 3 mL under vacuum and then kept stationary at room temperature over three days to induce the formation of X-ray quality yellow crystals of **7**. Mp: > 156 °C gradually melts and decomposes. ^{1}H NMR (400.22 MHz, THF-d$_8$): δ 1.18–1.38 [m, 60H, C(C*H*$_3$)$_3$ and CH(C*H*$_3$)$_2$], 2.82–3.05 [m, 4H, C*H*(CH$_3$)$_2$], 7.23–7.49 (m, 16H, Ar-*H*). ^{13}C{^{1}H} NMR (100.65 MHz, THF-d$_8$): δ 24.53, 24.76, 24.85, 24.97 [CH(*C*H$_3$)$_2$], 29.78, 29.95 [*C*H(CH$_3$)$_2$], 32.26, 32.43, 33.40 [C(*C*H$_3$)$_3$], 54.72, 55.11, 56.73 [*C*(CH$_3$)$_3$], 120.40 (S–C=C–S), 124.36, 124.42, 126.06, 128.71, 128.89, 128.97, 129.71, 129.96, 130.06, 130.28, 131.01, 131.05, 131.57, 134.97, 135.23, 135.46, 148.09, 148.20 (Ar-*C*), 158.62, 165.83 [N*C*(Ph)N], 169.17 (C=S). ^{29}Si{^{1}H} NMR (79.51 MHz, THF-d$_8$): δ 17.58, -51.66. Crystal data for **7**: $C_{57}H_{80}N_6S_3Si_2$, fw = 1001.63, triclinic, *P*-1 (No. 2), a = 11.5895(8) Å, b = 14.4609(10) Å, c = 18.9312(12) Å, α = 71.923(2)°, β = 73.303(2)°, γ = 76.552(2)°, V = 2852.9(3) Å^3, Z = 2, R1 = 0.0683 for 6080 data ($I > 2\sigma(I)$), wR$_2$ = 0.1391 (all data).

Compound **5**

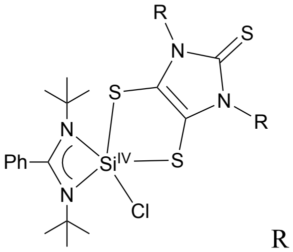

R = 2,6-diisopropylphenyl

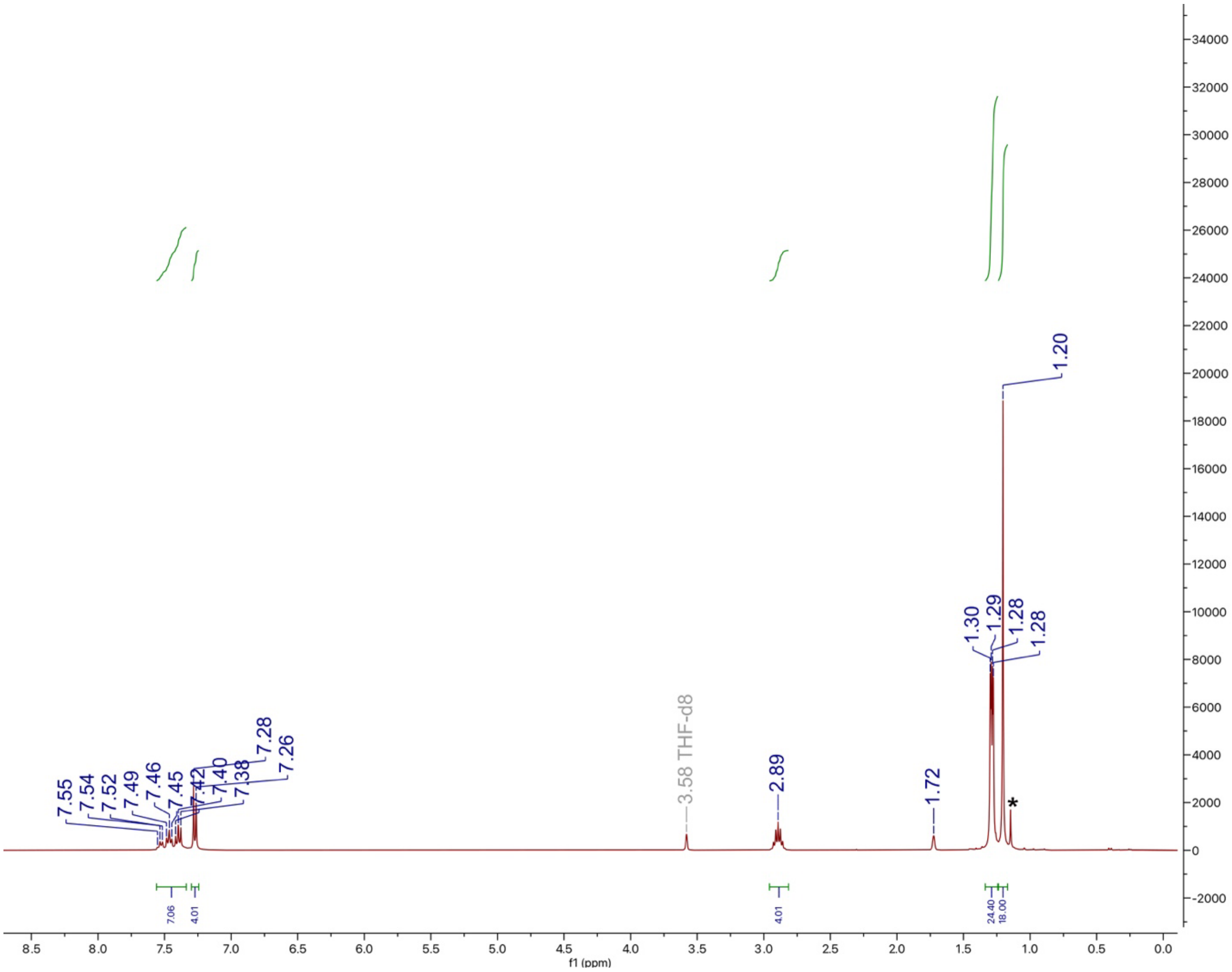

Figure S1. ^{1}H NMR spectrum of **5** in THF-d_8 (*: impurity).

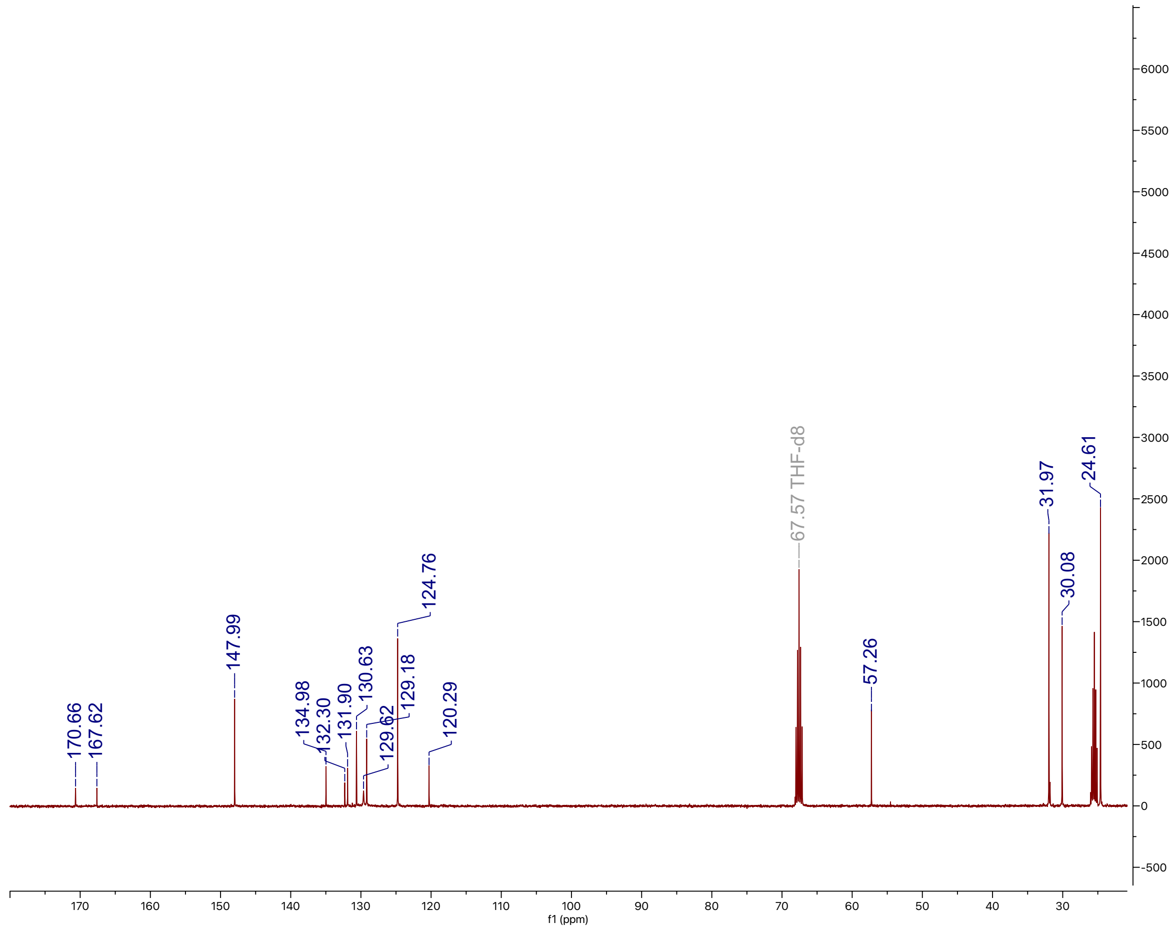

Figure S2. $^{13}C\{^1H\}$ NMR spectrum of **5** in THF-d_8.

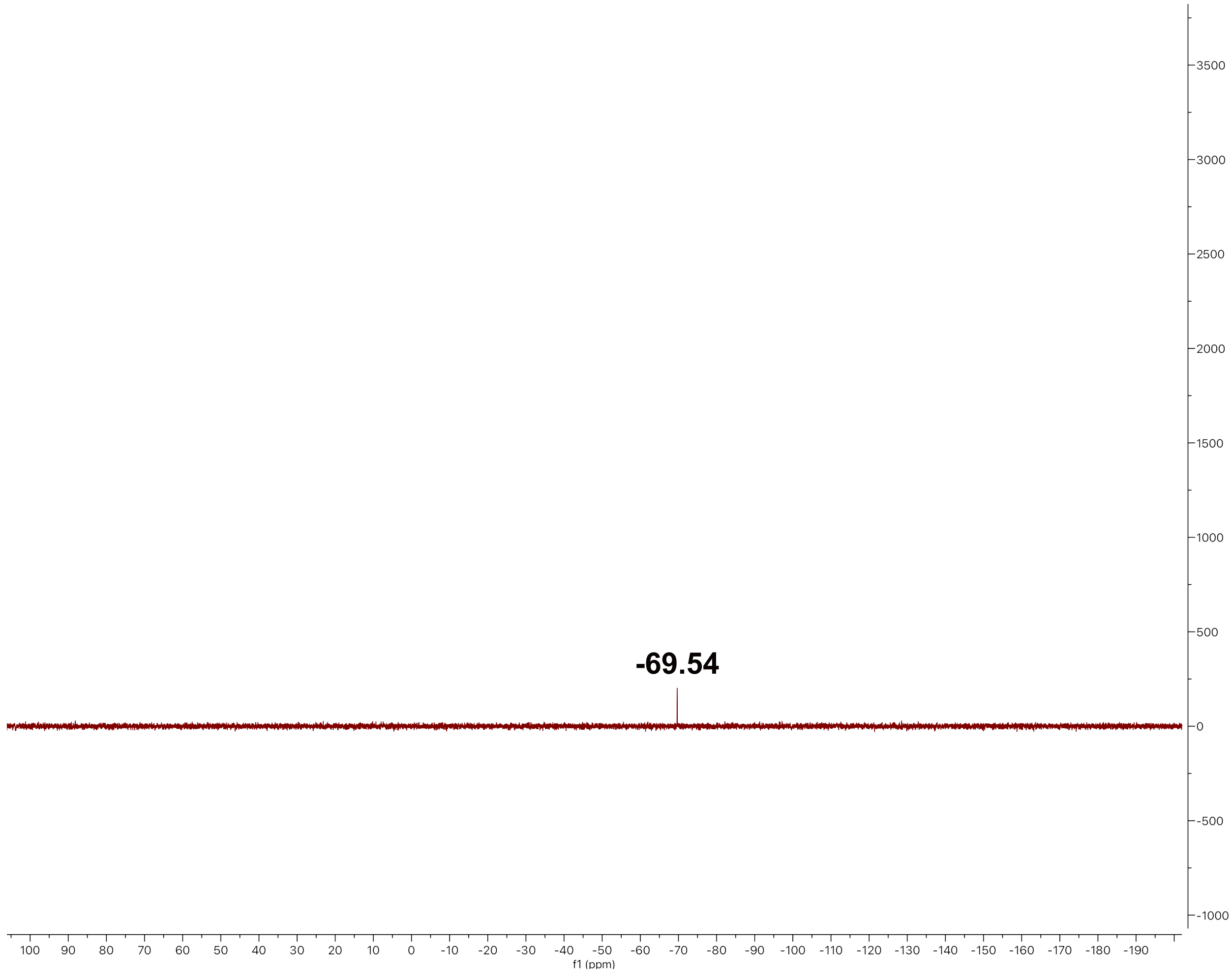

Figure S3. $^{29}Si\{^{1}H\}$ NMR spectrum of **5** in THF-d_8.

Compound **6**

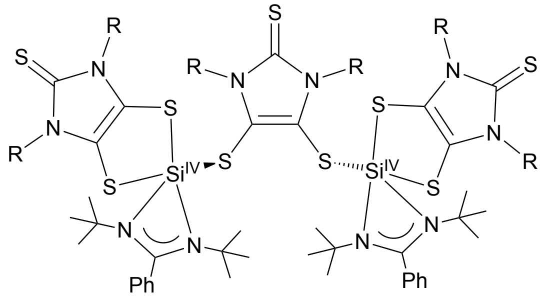

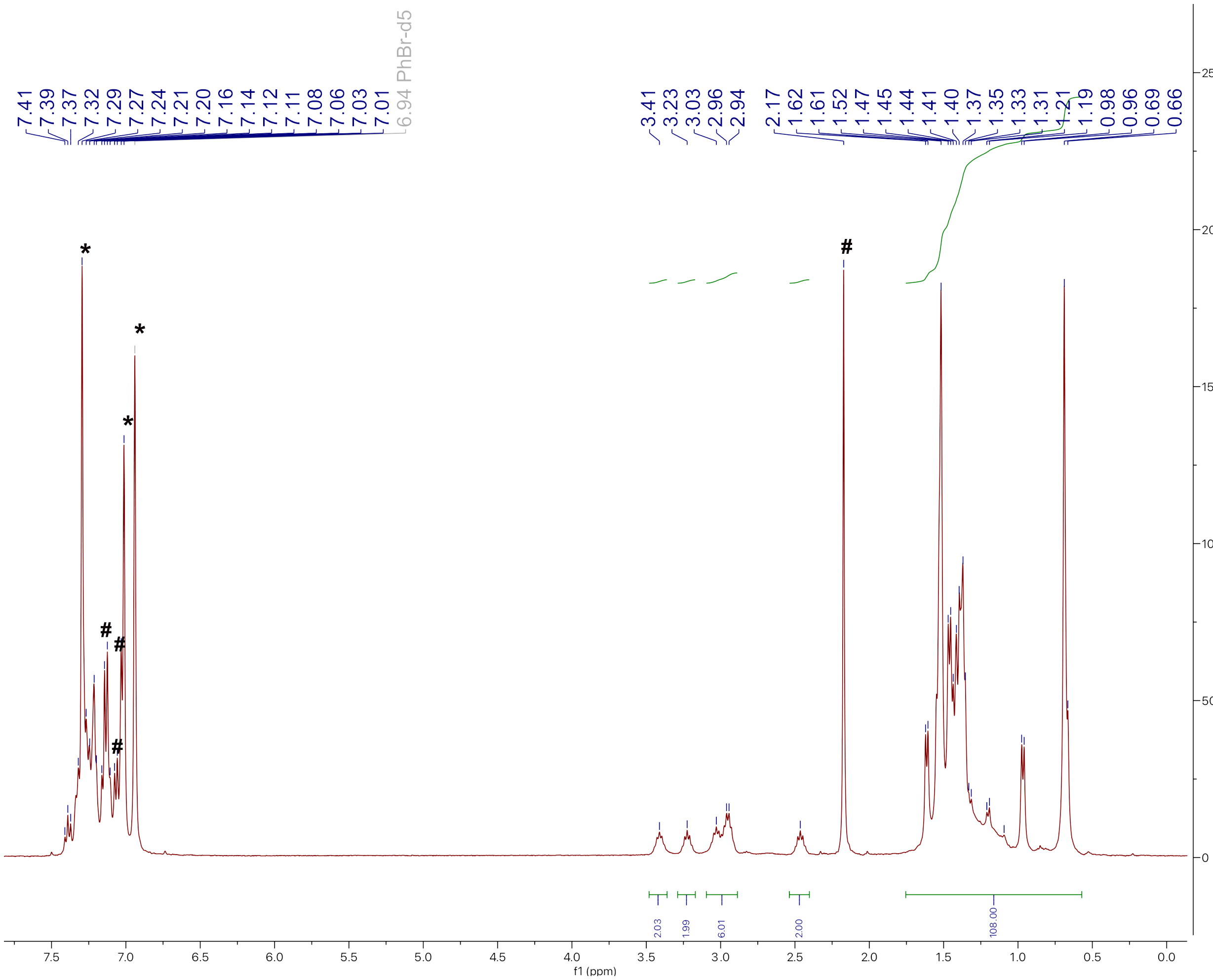

Figure S4. ^{1}H NMR spectrum of **6** in PhBr-d$_5$ [*: PhBr-d$_5$ (6.94, 7.01, and 7.29 ppm); $^{\#}$: toluene (2.17, 7.01, 7.03, 7.05, 7.07, 7.12, 7.14, and 7.16 ppm)].

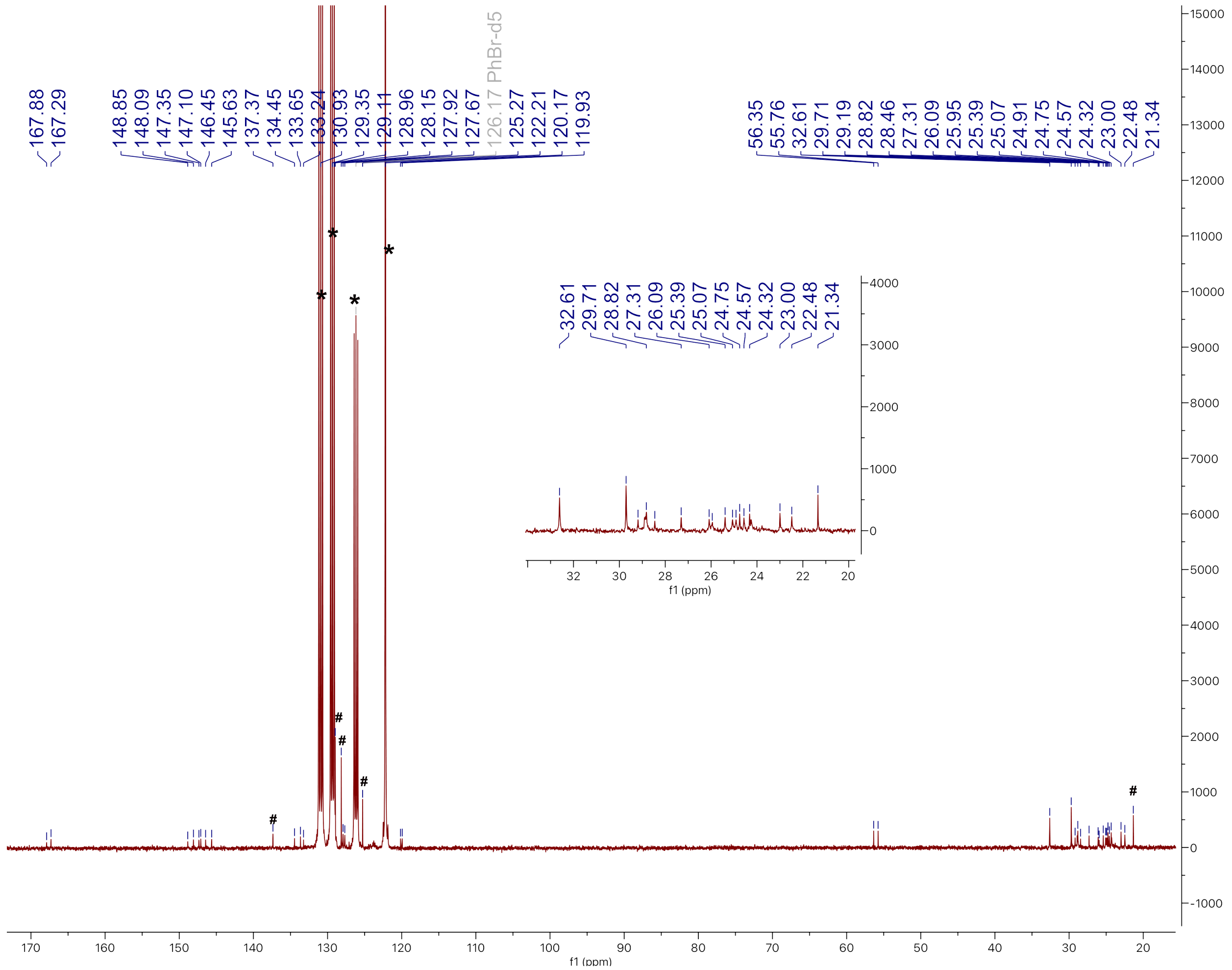

Figure S5. $^{13}C\{^1H\}$ NMR spectrum of **6** in PhBr-d_5 [*: PhBr-d_5 (122.21, 126.17(t), 129.35(t), and 130.93(t) ppm); $^{\#}$: toluene (21.34, 125.27, 128.15, 128.96, and 137.37 ppm)].

.

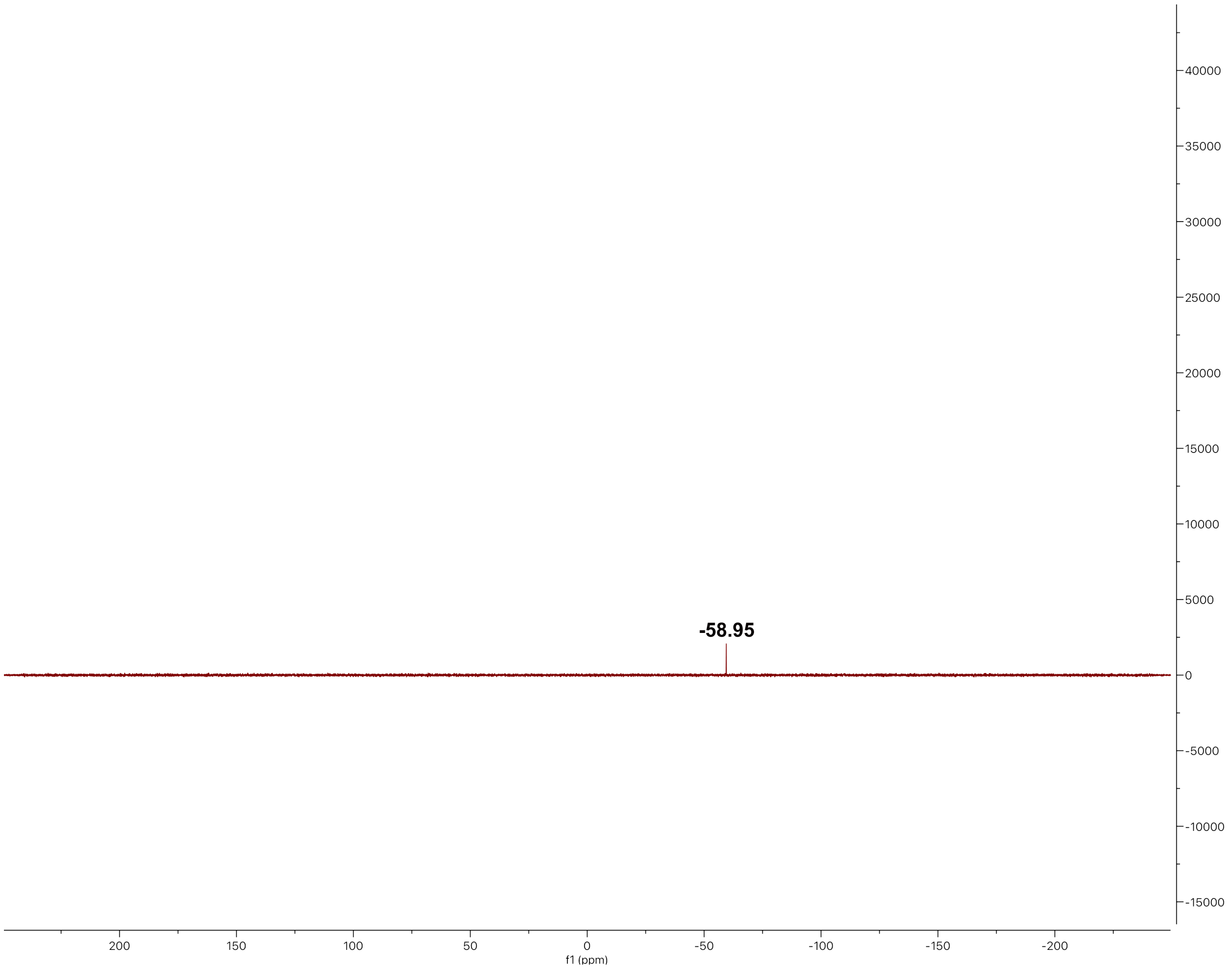

Figure S6. $^{29}Si\{^{1}H\}$ NMR spectrum of **6** in PhBr-d_5.

Compound **7**

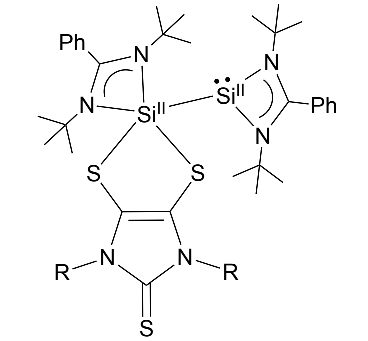

(R = 2,6-diisopropylphenyl)

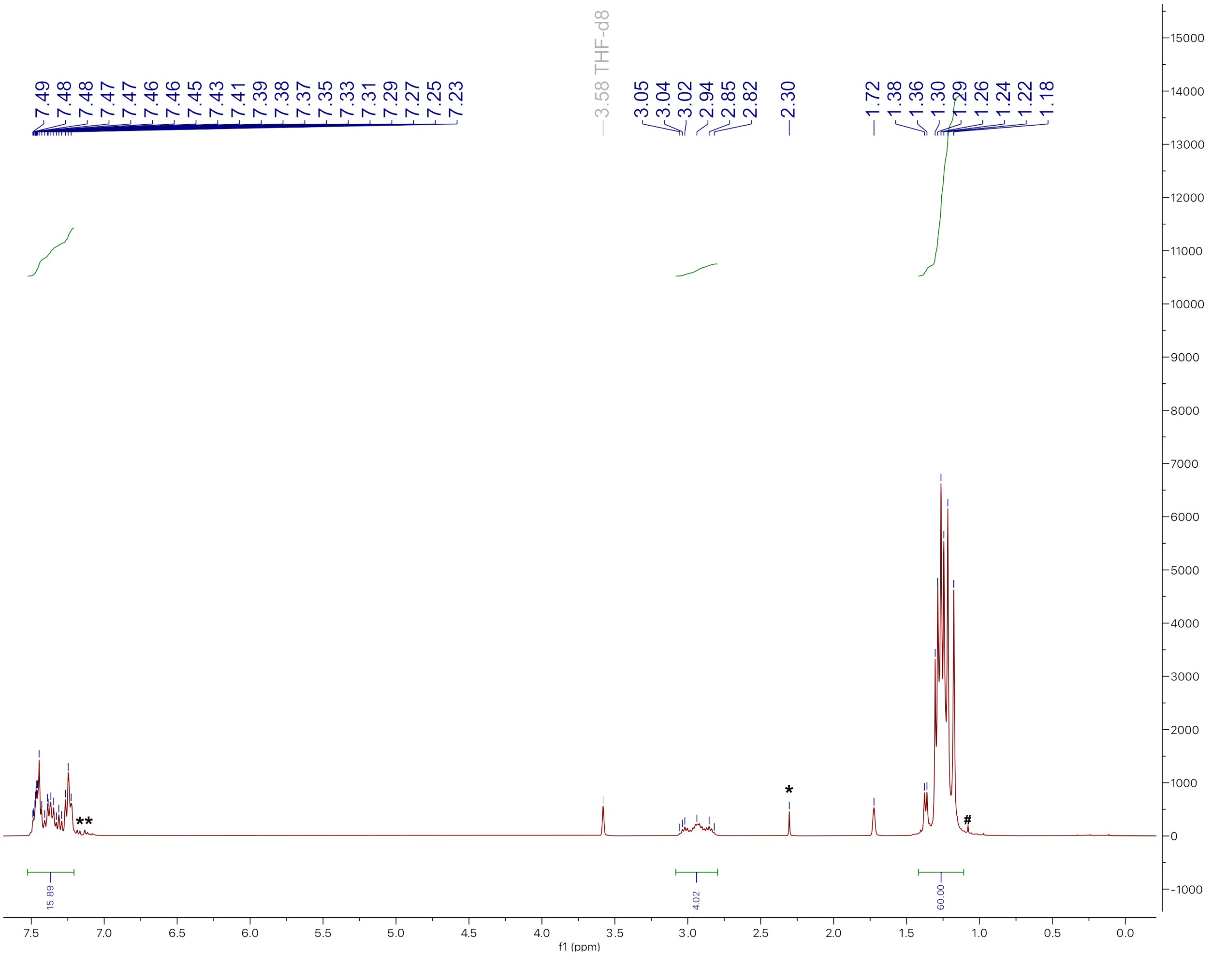

Fig. S7. ^{1}H NMR spectrum of **7** in THF-d_8 (*: resonances of toluene; #: impurity).

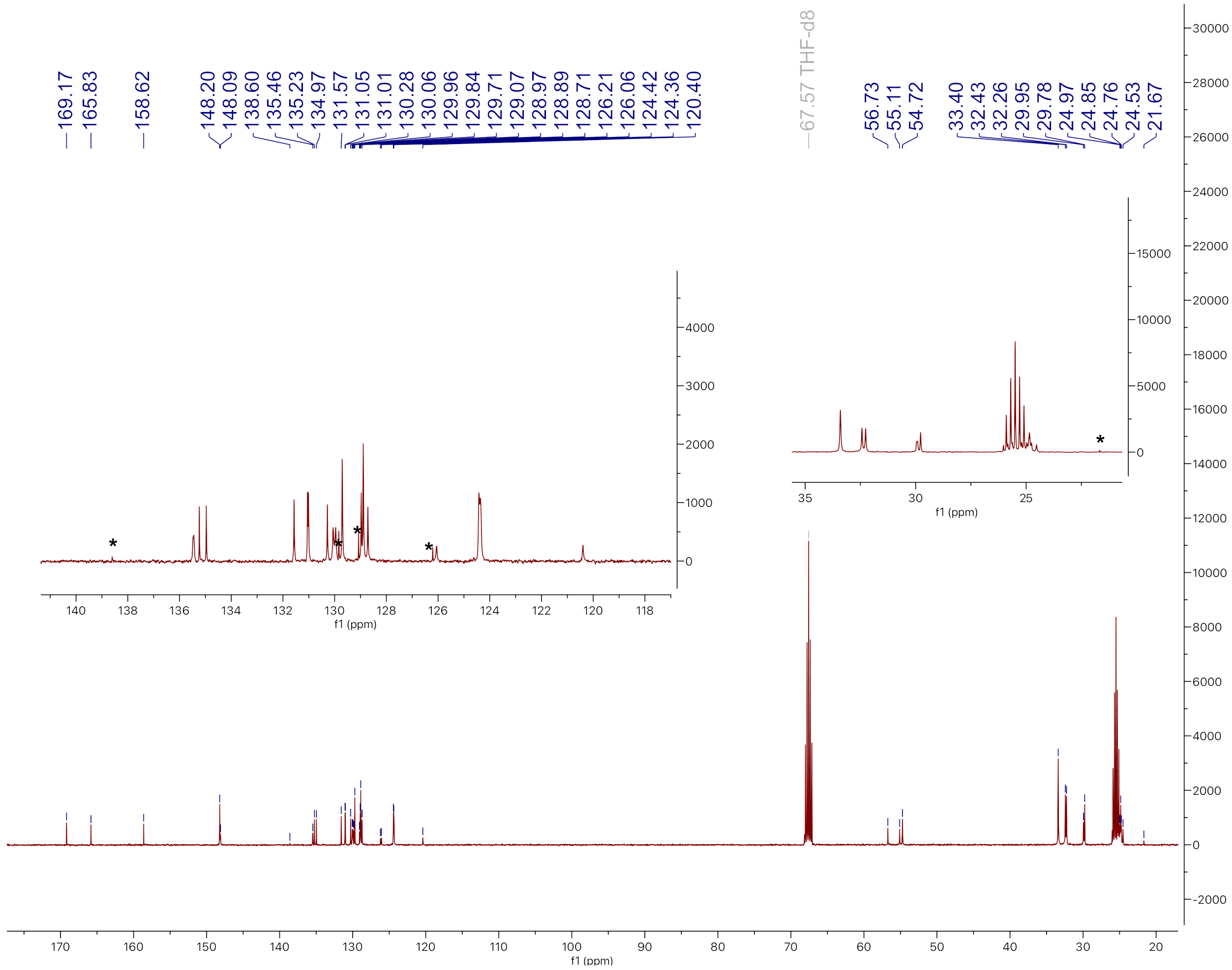

Fig. S8. $^{13}C\{^1H\}$ NMR spectrum of **7** in THF-d_8 (*: resonances of toluene).

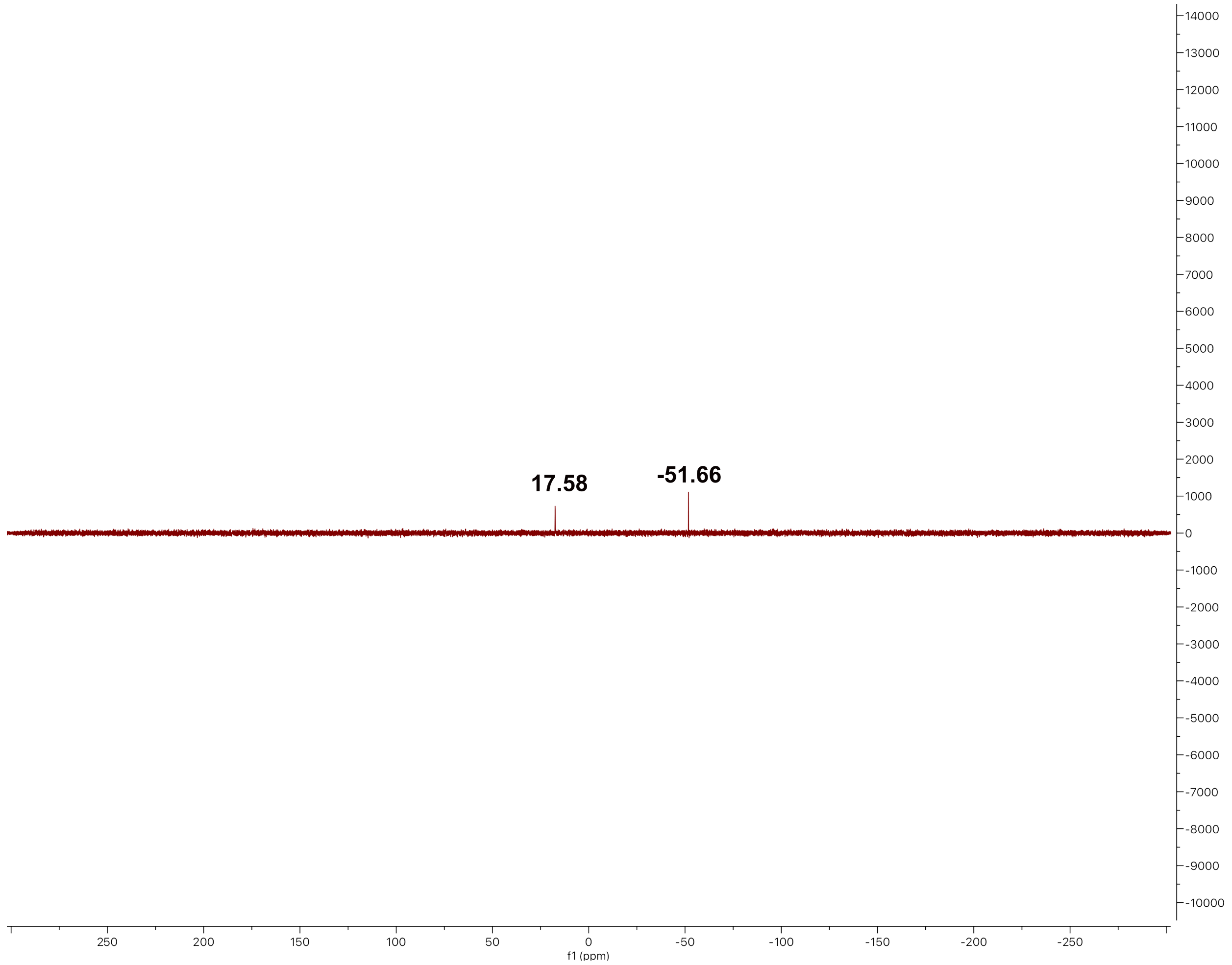

Fig. S9. $^{29}Si\{^1H\}$ NMR spectrum of **7** in THF-d_8.

SUPPORTING INFORMATION of COMPUTATIONS

All computations employed the Gaussian 16 (Revision C.01) program:

Gaussian 16, Revision C.01,
M. J. Frisch, G. W. Trucks, H. B. Schlegel, G. E. Scuseria,
M. A. Robb, J. R. Cheeseman, G. Scalmani, V. Barone,
G. A. Petersson, H. Nakatsuji, X. Li, M. Caricato, A. V. Marenich,
J. Bloino, B. G. Janesko, R. Gomperts, B. Mennucci, H. P. Hratchian,
J. V. Ortiz, A. F. Izmaylov, J. L. Sonnenberg, D. Williams-Young,
F. Ding, F. Lipparini, F. Egidi, J. Goings, B. Peng, A. Petrone,
T. Henderson, D. Ranasinghe, V. G. Zakrzewski, J. Gao, N. Rega,
G. Zheng, W. Liang, M. Hada, M. Ehara, K. Toyota, R. Fukuda,
J. Hasegawa, M. Ishida, T. Nakajima, Y. Honda, O. Kitao, H. Nakai,
T. Vreven, K. Throssell, J. A. Montgomery, Jr., J. E. Peralta,
F. Ogliaro, M. J. Bearpark, J. J. Heyd, E. N. Brothers, K. N. Kudin,
V. N. Staroverov, T. A. Keith, R. Kobayashi, J. Normand,
K. Raghavachari, A. P. Rendell, J. C. Burant, S. S. Iyengar,
J. Tomasi, M. Cossi, J. M. Millam, M. Klene, C. Adamo, R. Cammi,
J. W. Ochterski, R. L. Martin, K. Morokuma, O. Farkas,
J. B. Foresman, and D. J. Fox, Gaussian, Inc., Wallingford CT, 2019.

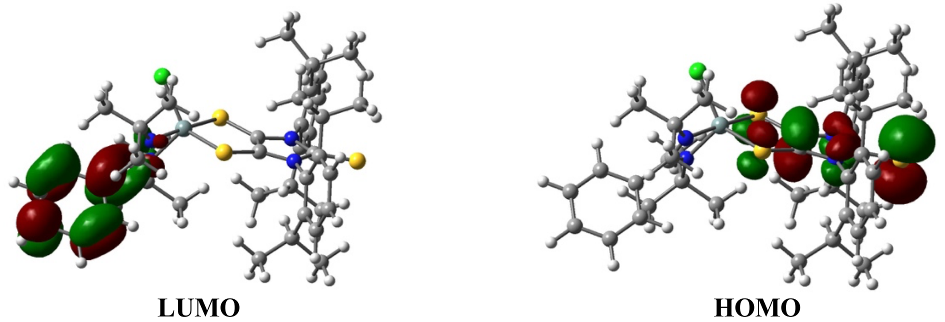

(a)

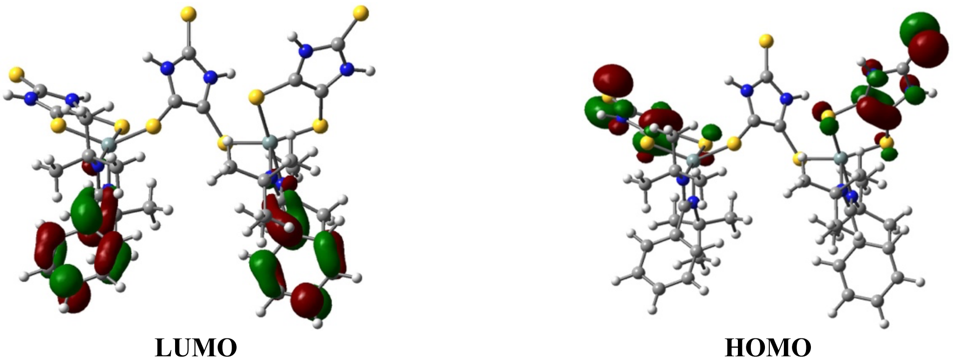

(b)

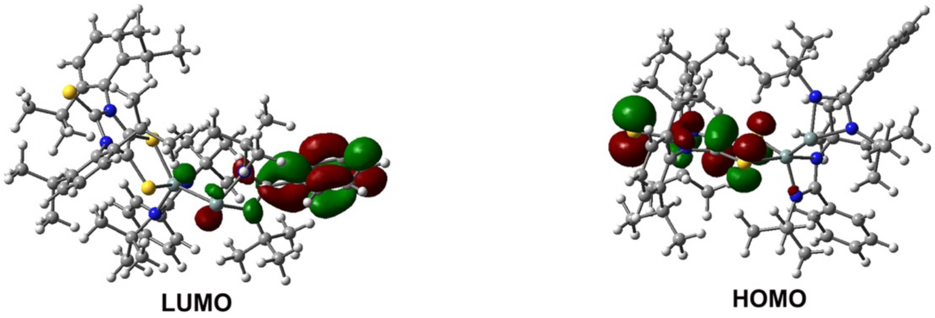

(c)

Fig. S10. (a) LUMO and HOMO of **5**; (b) LUMO and HOMO of **6-H**; (c) LUMO and HOMO of **7**.

SUPPORTING INFORMATION of X-RAY

Compound **5**

Table S1. Sample and crystal data for **5**.

**Identification code**	**5**	
**Chemical formula**	$C_{42}H_{57}ClN_4S_3Si$	
**Formula weight**	777.63 g/mol	
**Temperature**	135(2) K	
**Wavelength**	0.71073 Å	
**Crystal size**	0.100 x 0.160 x 0.240 mm	
**Crystal system**	triclinic	
**Space group**	P -1 (No. 2)	
**Unit cell dimensions**	a = 11.8139(18) Å	α = 80.716(4)°
	b = 13.715(2) Å	β = 79.422(4)°
	c = 14.983(2) Å	γ = 69.015(4)°
**Volume**	2216.1(6) $Å^3$	
**Z**	2	
**Density (calculated)**	1.165 g/cm^3	
**Absorption coefficient**	0.287 mm^{-1}	
**F(000)**	832	

Table S2. Data collection and structure refinement for **5**.

**Theta range for data collection**	1.86 to 27.50°	
**Index ranges**	-15<=h<=15, -17<=k<=17, -19<=l<=19	
**Reflections collected**	59763	
**Independent reflections**	10172 [R(int) = 0.1565]	
**Max. and min. transmission**	0.7460 and 0.4444	
**Structure solution technique**	direct methods	
**Structure solution program**	SHELXT 2014/5 (Sheldrick, 2014)	
**Refinement method**	Full-matrix least-squares on F^2	
**Refinement program**	SHELXL-2018/3 (Sheldrick, 2018)	
**Function minimized**	Σ w(F_o^2 - F_c^2)^2	
**Data / restraints / parameters**	10172 / 0 / 460	
**Goodness-of-fit on F^2**	1.018	
**Final R indices**	5986 data; I>2σ(I)	R1 = 0.0946, wR2 = 0.2057
	all data	R1 = 0.1588, wR2 = 0.2429
**Weighting scheme**	$w=1/[\sigma^2(F_o^2)+(0.0879P)^2+8.6722P]$ where $P=(F_o^2+2F_c^2)/3$	
**Largest diff. peak and hole**	1.150 and -0.589 eÅ^{-3}	
**R.M.S. deviation from mean**	0.102 eÅ^{-3}	

Table S3. Bond lengths (Å) for **5**.

Si1-N4	1.805(4)	Si1-N3	1.906(4)
Si1-Cl1	2.0921(19)	Si1-S2	2.1717(18)
Si1-S3	2.2498(18)	S1-C1	1.666(5)
S2-C2	1.722(5)	S3-C3	1.727(4)
N1-C1	1.354(6)	N1-C2	1.396(6)
N1-C16	1.440(5)	N2-C1	1.379(6)
N2-C3	1.392(6)	N2-C4	1.433(6)
N3-C28	1.322(6)	N3-C35	1.491(6)
N4-C28	1.349(6)	N4-C39	1.496(6)
C2-C3	1.333(6)	C4-C9	1.386(7)
C4-C5	1.392(7)	C5-C6	1.385(7)
C5-C13	1.514(8)	C6-C7	1.380(8)
C7-C8	1.380(8)	C8-C9	1.383(7)
C9-C10	1.518(7)	C10-C12	1.526(8)
C10-C11	1.525(8)	C13-C15	1.522(8)
C13-C14	1.528(8)	C16-C21	1.390(6)
C16-C17	1.398(6)	C17-C18	1.388(7)
C17-C25	1.512(7)	C18-C19	1.377(7)
C19-C20	1.382(7)	C20-C21	1.376(6)
C21-C22	1.522(7)	C22-C24	1.524(7)
C22-C23	1.525(8)	C25-C27	1.521(8)
C25-C26	1.528(8)	C28-C29	1.492(6)
C29-C30	1.381(7)	C29-C34	1.388(7)
C30-C31	1.387(7)	C31-C32	1.385(7)
C32-C33	1.374(7)	C33-C34	1.377(7)
C35-C36	1.527(7)	C35-C38	1.517(7)
C35-C37	1.527(7)	C39-C42	1.525(7)
C39-C40	1.532(7)	C39-C41	1.521(7)

Table S4. Bond angles (°) for **5**.

N4-Si1-N3	70.50(18)	N4-Si1-Cl1	111.66(14)
N3-Si1-Cl1	92.71(14)	N4-Si1-S2	113.35(14)
N3-Si1-S2	84.56(13)	Cl1-Si1-S2	130.90(8)
N4-Si1-S3	106.21(14)	N3-Si1-S3	176.23(15)
Cl1-Si1-S3	90.26(7)	S2-Si1-S3	95.25(7)
C2-S2-Si1	98.57(16)	C3-S3-Si1	97.02(16)
C1-N1-C2	110.5(4)	C1-N1-C16	125.8(4)
C2-N1-C16	123.1(4)	C1-N2-C3	109.7(4)
C1-N2-C4	124.7(4)	C3-N2-C4	125.5(4)
C28-N3-C35	129.4(4)	C28-N3-Si1	89.5(3)
C35-N3-Si1	140.8(3)	C28-N4-C39	129.9(4)
C28-N4-Si1	93.0(3)	C39-N4-Si1	135.8(3)
N1-C1-N2	105.0(4)	N1-C1-S1	128.2(3)
N2-C1-S1	126.9(3)	C3-C2-N1	107.3(4)
C3-C2-S2	124.7(4)	N1-C2-S2	127.9(3)
C2-C3-N2	107.5(4)	C2-C3-S3	123.5(4)
N2-C3-S3	128.8(3)	C9-C4-C5	122.4(4)
C9-C4-N2	118.8(4)	C5-C4-N2	118.8(4)
C4-C5-C6	117.6(5)	C4-C5-C13	122.3(4)
C6-C5-C13	120.1(5)	C5-C6-C7	121.3(5)
C8-C7-C6	119.4(5)	C7-C8-C9	121.3(5)
C4-C9-C8	117.9(5)	C4-C9-C10	121.6(4)
C8-C9-C10	120.4(5)	C9-C10-C12	110.6(5)
C9-C10-C11	111.7(5)	C12-C10-C11	111.6(5)
C5-C13-C15	111.4(5)	C5-C13-C14	111.7(5)
C15-C13-C14	110.9(5)	C21-C16-C17	123.2(4)
C21-C16-N1	119.3(4)	C17-C16-N1	117.5(4)
C18-C17-C16	116.7(4)	C18-C17-C25	120.9(4)
C16-C17-C25	122.4(4)	C17-C18-C19	121.4(5)
C20-C19-C18	119.9(4)	C19-C20-C21	121.3(5)
C16-C21-C20	117.4(4)	C16-C21-C22	120.5(4)
C20-C21-C22	122.1(4)	C21-C22-C24	111.0(5)
C21-C22-C23	113.5(4)	C24-C22-C23	110.0(4)
C17-C25-C27	112.7(4)	C17-C25-C26	110.3(4)
C27-C25-C26	111.5(5)	N3-C28-N4	106.7(4)
N3-C28-C29	126.9(4)	N4-C28-C29	126.1(4)
C30-C29-C34	120.3(4)	C30-C29-C28	118.2(4)
C34-C29-C28	121.5(4)	C29-C30-C31	119.9(5)
C32-C31-C30	119.2(5)	C33-C32-C31	120.8(5)
C32-C33-C34	120.1(5)	C33-C34-C29	119.6(5)
N3-C35-C36	108.6(4)	N3-C35-C38	107.4(4)
C36-C35-C38	109.3(5)	N3-C35-C37	111.8(4)
C36-C35-C37	111.0(4)	C38-C35-C37	108.6(5)
N4-C39-C42	108.3(4)	N4-C39-C40	113.6(4)
C42-C39-C40	109.6(4)	N4-C39-C41	108.0(4)
C42-C39-C41	111.9(5)	C40-C39-C41	105.6(4)

Compound **6**

Table S5. Sample and crystal data for **6**.

**Identification code**	**6**	
**Chemical formula**	$C_{111}H_{148}N_{10}S_9Si_2$	
**Formula weight**	1967.11 g/mol	
**Temperature**	135(2) K	
**Wavelength**	0.71073 Å	
**Crystal size**	0.090 x 0.150 x 0.240 mm	
**Crystal system**	monoclinic	
**Space group**	$P2_1/n$ (No. 14)	
**Unit cell dimensions**	a = 24.3272(18) Å	α = 90°
	b = 23.1614(18) Å	β = 105.439(2)°
	c = 27.091(2) Å	γ = 90°
**Volume**	14714(2) $Å^3$	
**Z**	4	
**Density (calculated)**	0.888 g/cm^3	
**Absorption coefficient**	0.190 mm^{-1}	
**F(000)**	4224	

Table S6. Data collection and structure refinement for **6**.

**Theta range for data collection**	1.18 to 25.25°	
**Index ranges**	-29<=h<=28, -27<=k<=27, -15<=l<=32	
**Reflections collected**	26617	
**Coverage of independent reflections**	99.9%	
**Absorption correction**	Multi-Scan	
**Max. and min. transmission**	0.7454 and 0.6351	
**Structure solution technique**	direct methods	
**Structure solution program**	SHELXT 2014/5 (Sheldrick, 2014)	
**Refinement method**	Full-matrix least-squares on F^2	
**Refinement program**	SHELXL-2018/3 (Sheldrick, 2018)	
**Function minimized**	$\Sigma\, w(F_o^2 - F_c^2)^2$	
**Data / restraints / parameters**	26617 / 0 / 1190	
**Goodness-of-fit on F^2**	1.080	
**Δ/σ_{max}**	0.001	
**Final R indices**	15855 data; $I>2\sigma(I)$	R1 = 0.1034, wR2 = 0.2182
	all data	R1 = 0.1652, wR2 = 0.2444
**Weighting scheme**	$w=1/[\sigma^2(F_o^2)+(0.0338P)^2+91.8417P]$ where $P=(F_o^2+2F_c^2)/3$	
**Largest diff. peak and hole**	0.558 and -0.687 eÅ^{-3}	
**R.M.S. deviation from mean**	0.099 eÅ^{-3}	

Table S7. Bond lengths (Å) for **6**.

Si1-N7	1.823(5)	Si1-N8	1.933(5)
Si1-S5	2.175(2)	Si1-S3	2.200(2)
Si1-S2	2.258(2)	Si2-N10	1.811(5)
Si2-N9	1.920(5)	Si2-S6	2.174(2)
Si2-S9	2.196(2)	Si2-S8	2.255(2)
S1-C1	1.652(7)	S2-C2	1.739(6)
S3-C3	1.723(6)	S4-C28	1.671(6)
S5-C29	1.740(6)	S6-C30	1.738(6)
S7-C55	1.668(7)	S9-C56	1.733(6)
S8-C57	1.753(6)	N1-C2	1.414(7)
N1-C1	1.387(8)	N1-C16	1.437(8)
N2-C1	1.377(8)	N2-C3	1.398(7)
N2-C4	1.420(8)	N3-C28	1.361(7)
N3-C43	1.441(7)	N3-C29	1.414(7)
N4-C28	1.358(7)	N4-C31	1.434(7)
N4-C30	1.424(7)	N6-C55	1.373(8)
N6-C56	1.395(8)	N6-C70	1.450(8)
N5-C55	1.364(8)	N5-C57	1.427(7)
N5-C58	1.455(8)	N7-C82	1.348(8)
N7-C83	1.507(8)	N8-C82	1.307(8)
N8-C87	1.489(7)	N9-C97	1.327(7)
N9-C98	1.507(8)	N10-C97	1.365(7)
N10-C03	1.483(8)	C2-C3	1.339(8)
C4-C5	1.390(10)	C4-C9	1.411(9)
C5-C6	1.378(9)	C5-C13	1.526(10)
C6-C7	1.390(10)	C7-C8	1.345(10)
C8-C9	1.409(9)	C9-C10	1.504(10)
C10-C11	1.517(10)	C10-C12	1.532(11)
C13-C14	1.519(11)	C13-C15	1.532(11)
C16-C21	1.389(9)	C16-C17	1.407(9)
C17-C18	1.401(9)	C17-C25	1.516(10)
C18-C19	1.343(10)	C19-C20	1.362(10)
C20-C21	1.390(9)	C21-C22	1.516(10)
C22-C24	1.542(10)	C22-C23	1.564(11)
C25-C26	1.511(10)	C25-C27	1.531(11)
C29-C30	1.343(7)	C31-C36	1.379(8)
C31-C32	1.419(8)	C32-C33	1.407(8)

C32-C40	1.491(9)	C33-C34	1.372(9)
C34-C35	1.373(9)	C35-C36	1.401(8)
C36-C37	1.533(8)	C37-C38	1.498(9)
C37-C39	1.564(9)	C40-C42	1.535(10)
C40-C41	1.542(9)	C43-C44	1.385(8)
C43-C48	1.421(8)	C44-C45	1.407(8)
C44-C52	1.514(9)	C45-C46	1.375(9)
C46-C47	1.368(9)	C47-C48	1.401(8)
C48-C49	1.498(8)	C49-C51	1.544(9)
C49-C50	1.544(9)	C52-C53	1.543(9)
C52-C54	1.525(9)	C56-C57	1.312(8)
C58-C63	1.381(10)	C58-C59	1.390(10)
C59-C60	1.398(10)	C59-C67	1.514(11)
C60-C61	1.331(11)	C61-C62	1.376(11)
C62-C63	1.382(10)	C63-C64	1.529(10)
C64-C65	1.505(11)	C64-C66	1.567(10)
C67-C68	1.512(11)	C67-C69	1.544(12)
C70-C71	1.399(10)	C70-C75	1.386(10)
C71-C72	1.396(10)	C71-C79	1.484(12)
C72-C73	1.380(12)	C73-C74	1.374(12)
C74-C75	1.394(10)	C75-C76	1.542(11)
C76-C78	1.489(10)	C76-C77	1.531(10)
C79-C80	1.511(12)	C79-C81	1.520(15)
C82-C91	1.502(9)	C83-C84	1.513(9)
C83-C85	1.541(9)	C83-C86	1.525(9)
C87-C90	1.517(10)	C87-C89	1.527(10)
C87-C88	1.560(9)	C91-C96	1.386(10)
C91-C92	1.405(10)	C92-C93	1.366(11)
C93-C94	1.325(14)	C94-C95	1.389(13)
C95-C96	1.370(11)	C97-C07	1.477(8)
C98-C99	1.506(9)	C98-C01	1.526(9)
C98-C02	1.535(9)	C03-C06	1.516(9)
C03-C05	1.535(9)	C03-C04	1.551(9)
C07-C012	1.388(9)	C07-C08	1.390(9)
C08-C09	1.373(9)	C09-C010	1.381(11)
C010-C011	1.387(11)	C011-C012	1.368(10)

Table S8. Bond angles (°) for **6**.

N7-Si1-N8	69.5(2)	N7-Si1-S5	108.98(17)
N8-Si1-S5	84.31(16)	N7-Si1-S3	105.37(17)
N8-Si1-S3	84.30(16)	S5-Si1-S3	137.07(10)
N7-Si1-S2	110.79(17)	N8-Si1-S2	178.88(17)
S5-Si1-S2	96.56(8)	S3-Si1-S2	94.58(8)
N10-Si2-N9	70.5(2)	N10-Si2-S6	109.63(17)
N9-Si2-S6	83.49(16)	N10-Si2-S9	104.65(17)
N9-Si2-S9	83.98(16)	S6-Si2-S9	136.80(10)
N10-Si2-S8	110.08(17)	N9-Si2-S8	178.85(18)
S6-Si2-S8	97.23(8)	S9-Si2-S8	94.89(8)
C2-S2-Si1	96.2(2)	C3-S3-Si1	97.5(2)
C29-S5-Si1	114.4(2)	C30-S6-Si2	114.64(19)
C56-S9-Si2	96.0(2)	C57-S8-Si2	95.4(2)
C2-N1-C1	109.4(5)	C2-N1-C16	129.4(5)
C1-N1-C16	120.8(5)	C1-N2-C3	109.9(5)
C1-N2-C4	124.6(5)	C3-N2-C4	125.4(5)
C28-N3-C43	126.0(5)	C28-N3-C29	109.7(4)
C43-N3-C29	123.2(4)	C28-N4-C31	125.8(5)
C28-N4-C30	109.3(4)	C31-N4-C30	123.6(4)
C55-N6-C56	109.1(5)	C55-N6-C70	126.2(5)
C56-N6-C70	124.7(5)	C55-N5-C57	108.7(5)
C55-N5-C58	123.9(5)	C57-N5-C58	126.8(5)
C82-N7-C83	131.0(5)	C82-N7-Si1	93.0(4)
C83-N7-Si1	135.2(4)	C82-N8-C87	130.2(5)
C82-N8-Si1	89.5(4)	C87-N8-Si1	140.1(4)
C97-N9-C98	129.2(5)	C97-N9-Si2	89.4(4)
C98-N9-Si2	140.9(4)	C97-N10-C03	129.3(5)
C97-N10-Si2	93.0(4)	C03-N10-Si2	135.9(4)
N2-C1-N1	105.2(5)	N2-C1-S1	127.0(5)
N1-C1-S1	127.7(5)	C3-C2-N1	107.3(5)
C3-C2-S2	123.2(5)	N1-C2-S2	129.5(5)
C2-C3-N2	108.2(5)	C2-C3-S3	124.8(5)
N2-C3-S3	126.6(4)	C5-C4-C9	122.1(6)
C5-C4-N2	119.2(6)	C9-C4-N2	118.7(6)
C4-C5-C6	117.6(7)	C4-C5-C13	122.1(6)
C6-C5-C13	120.3(7)	C5-C6-C7	122.2(7)
C8-C7-C6	119.0(6)	C7-C8-C9	122.6(7)
C4-C9-C8	116.4(7)	C4-C9-C10	122.7(6)
C8-C9-C10	120.8(6)	C9-C10-C11	112.3(6)
C9-C10-C12	112.0(6)	C11-C10-C12	110.9(7)
C5-C13-C14	112.4(7)	C5-C13-C15	112.1(7)
C14-C13-C15	108.4(7)	C21-C16-C17	121.9(6)
C21-C16-N1	119.1(6)	C17-C16-N1	118.9(6)

C16-C17-C18	116.3(7)	C16-C17-C25	122.0(6)
C18-C17-C25	121.7(7)	C19-C18-C17	122.2(7)
C18-C19-C20	120.6(7)	C21-C20-C19	121.1(7)
C16-C21-C20	117.9(6)	C16-C21-C22	123.6(6)
C20-C21-C22	118.3(7)	C21-C22-C24	111.8(7)
C21-C22-C23	110.5(6)	C24-C22-C23	109.9(7)
C17-C25-C26	114.4(6)	C17-C25-C27	110.3(7)
C26-C25-C27	110.4(7)	N4-C28-N3	106.6(5)
N4-C28-S4	126.0(5)	N3-C28-S4	127.3(4)
C30-C29-N3	107.2(5)	C30-C29-S5	129.8(4)
N3-C29-S5	122.0(4)	C29-C30-N4	107.2(5)
C29-C30-S6	130.3(5)	N4-C30-S6	121.5(4)
C36-C31-C32	122.6(5)	C36-C31-N4	120.6(5)
C32-C31-N4	116.7(5)	C31-C32-C33	116.3(5)
C31-C32-C40	122.8(5)	C33-C32-C40	120.8(6)
C34-C33-C32	121.6(6)	C35-C34-C33	120.2(6)
C34-C35-C36	121.1(6)	C31-C36-C35	118.0(6)
C31-C36-C37	123.5(5)	C35-C36-C37	118.5(6)
C36-C37-C38	111.5(5)	C36-C37-C39	108.9(5)
C38-C37-C39	110.5(5)	C32-C40-C42	109.8(6)
C32-C40-C41	111.7(6)	C42-C40-C41	111.3(6)
C44-C43-N3	120.5(5)	C44-C43-C48	123.4(5)
N3-C43-C48	116.0(5)	C43-C44-C45	116.6(6)
C43-C44-C52	123.5(6)	C45-C44-C52	119.9(6)
C46-C45-C44	121.5(6)	C45-C46-C47	120.7(6)
C46-C47-C48	121.4(6)	C47-C48-C43	116.4(5)
C47-C48-C49	120.9(5)	C43-C48-C49	122.6(5)
C48-C49-C51	110.5(5)	C48-C49-C50	112.2(5)
C51-C49-C50	109.0(6)	C44-C52-C53	110.3(5)
C44-C52-C54	110.0(6)	C53-C52-C54	111.0(6)
N6-C55-N5	106.1(5)	N6-C55-S7	126.8(5)
N5-C55-S7	127.0(5)	C57-C56-N6	108.8(5)
C57-C56-S9	125.6(5)	N6-C56-S9	125.7(5)
C56-C57-N5	107.4(5)	C56-C57-S8	123.1(5)
N5-C57-S8	129.1(4)	C63-C58-C59	122.4(6)
C63-C58-N5	118.5(6)	C59-C58-N5	119.0(6)
C58-C59-C60	116.5(7)	C58-C59-C67	122.8(6)
C60-C59-C67	120.6(7)	C61-C60-C59	122.8(8)
C60-C61-C62	119.2(7)	C63-C62-C61	121.9(8)
C58-C63-C62	117.2(7)	C58-C63-C64	123.7(6)
C62-C63-C64	119.1(7)	C65-C64-C63	112.2(6)
C65-C64-C66	110.3(7)	C63-C64-C66	109.0(6)
C59-C67-C68	115.8(7)	C59-C67-C69	109.9(8)
C68-C67-C69	111.3(8)	C71-C70-C75	123.0(6)

C71-C70-N6	120.1(7)	C75-C70-N6	116.8(6)
C70-C71-C72	116.2(8)	C70-C71-C79	123.2(7)
C72-C71-C79	120.6(8)	C73-C72-C71	122.3(8)
C72-C73-C74	119.4(8)	C73-C74-C75	121.1(8)
C74-C75-C70	118.0(7)	C74-C75-C76	118.6(7)
C70-C75-C76	123.5(6)	C78-C76-C77	111.8(7)
C78-C76-C75	111.7(7)	C77-C76-C75	109.0(6)
C71-C79-C80	112.4(9)	C71-C79-C81	113.4(8)
C80-C79-C81	111.3(8)	N8-C82-N7	107.6(5)
N8-C82-C91	125.7(6)	N7-C82-C91	126.2(6)
N7-C83-C84	113.4(5)	N7-C83-C85	107.9(5)
C84-C83-C85	109.3(5)	N7-C83-C86	107.0(5)
C84-C83-C86	108.7(5)	C85-C83-C86	110.5(5)
N8-C87-C90	109.5(5)	N8-C87-C89	111.2(5)
C90-C87-C89	111.7(6)	N8-C87-C88	107.7(5)
C90-C87-C88	108.4(6)	C89-C87-C88	108.2(6)
C96-C91-C92	119.8(6)	C96-C91-C82	119.2(6)
C92-C91-C82	121.0(6)	C91-C92-C93	118.6(8)
C94-C93-C92	121.3(10)	C93-C94-C95	121.6(9)
C94-C95-C96	119.0(8)	C95-C96-C91	119.7(8)
N9-C97-N10	106.3(5)	N9-C97-C07	126.0(6)
N10-C97-C07	126.8(6)	C99-C98-N9	108.5(5)
C99-C98-C01	110.1(6)	N9-C98-C01	108.3(5)
C99-C98-C02	108.0(6)	N9-C98-C02	111.1(5)
C01-C98-C02	110.9(6)	N10-C03-C06	106.8(5)
N10-C03-C05	109.8(5)	C06-C03-C05	111.3(6)
N10-C03-C04	112.9(5)	C06-C03-C04	107.2(5)
C05-C03-C04	108.8(5)	C012-C07-C08	120.1(6)
C012-C07-C97	124.7(6)	C08-C07-C97	115.1(6)
C09-C08-C07	120.3(7)	C010-C09-C08	118.7(7)
C09-C010-C011	121.6(7)	C012-C011-C010	119.3(8)
C07-C012-C011	119.9(7)		

Compound **7**

Table S9. Sample and crystal data for **7**.

**Identification code**	**7**	
**Chemical formula**	$C_{57}H_{80}N_6S_3Si_2$	
**Formula weight**	1001.63 g/mol	
**Temperature**	135(2) K	
**Wavelength**	0.71073 Å	
**Crystal size**	0.120 x 0.180 x 0.280 mm	
**Crystal system**	triclinic	
**Space group**	P -1 (No. 2)	
**Unit cell dimensions**	a = 11.5895(8) Å	α = 71.923(2)°
	b = 14.4609(10) Å	β = 73.303(2)°
	c = 18.9312(12) Å	γ = 76.552(2)°
**Volume**	2852.9(3) $Å^3$	
**Z**	2	
**Density (calculated)**	1.166 g/cm^3	
**Absorption coefficient**	0.213 mm^{-1}	
**F(000)**	1080	

Table S10. Data collection and structure refinement for **7**.

**Theta range for data collection**	2.12 to 25.49°	
**Index ranges**	-14<=h<=14, -17<=k<=17, -22<=l<=22	
**Reflections collected**	71436	
**Independent reflections**	10565 [R(int) = 0.1915]	
**Max. and min. transmission**	0.7452 and 0.5694	
**Structure solution technique**	direct methods	
**Structure solution program**	SHELXT 2014/5 (Sheldrick, 2014)	
**Refinement method**	Full-matrix least-squares on F^2	
**Refinement program**	SHELXL-2018/3 (Sheldrick, 2018)	
**Function minimized**	$\Sigma\ w(F_o^2 - F_c^2)^2$	
**Data / restraints / parameters**	10565 / 82 / 641	
**Goodness-of-fit on F^2**	1.006	
**Final R indices**	6080 data; $I>2\sigma(I)$	R1 = 0.0683, wR2 = 0.1145
	all data	R1 = 0.1518, wR2 = 0.1391
**Weighting scheme**	$w=1/[\sigma^2(F_o^2)+(0.0430P)^2+2.2956P]$ where $P=(F_o^2+2F_c^2)/3$	
**Largest diff. peak and hole**	0.474 and -0.418 eÅ^{-3}	
**R.M.S. deviation from mean**	0.073 eÅ^{-3}	

Table S11. Bond lengths (Å) for **7**.

Si1-N4	1.838(3)	Si1-N3	1.952(3)
Si1-S3	2.1987(13)	Si1-Si2	2.3966(14)
Si1-S2	2.4291(13)	Si2-N6	1.875(3)
Si2-N5	1.870(3)	S1-C1	1.673(4)
S2-C2	1.728(3)	S3-C3	1.725(3)
N1-C1	1.364(4)	N1-C2	1.402(4)
N1-C16	1.435(4)	N2-C1	1.363(4)
N2-C3	1.398(4)	N2-C4	1.445(4)
N3-C28	1.317(4)	N3-C35	1.485(4)
N4-C28	1.347(4)	N4-C39	1.498(4)
N5-C43	1.342(4)	N5-C50	1.476(4)
N6-C43	1.328(4)	N6-C54	1.479(4)
C2-C3	1.346(5)	C4-C9	1.397(5)
C4-C5	1.391(5)	C5-C6	1.388(5)
C5-C13	1.522(7)	C5-C13'	1.527(16)
C6-C7	1.375(5)	C7-C8	1.378(5)
C8-C9	1.391(5)	C9-C10	1.512(5)
C10-C12	1.516(5)	C10-C11	1.525(5)
C13-C15	1.518(9)	C13-C14	1.527(9)
C13'-C15'	1.522(16)	C13'-C14'	1.529(18)
C16-C21	1.392(5)	C16-C17	1.392(5)
C17-C18	1.387(5)	C17-C25	1.513(5)
C18-C19	1.368(5)	C19-C20	1.372(5)
C20-C21	1.395(5)	C21-C22	1.508(5)
C22-C23	1.531(5)	C22-C24	1.522(5)
C25-C27	1.510(6)	C25-C26	1.521(6)
C28-C29	1.485(5)	C29-C30	1.369(6)
C29-C34	1.392(5)	C30-C31	1.430(7)
C31-C32	1.388(8)	C32-C33	1.331(8)
C33-C34	1.357(6)	C35-C37	1.516(5)
C35-C38	1.523(5)	C35-C36	1.535(5)
C39-C42	1.518(5)	C39-C40	1.521(5)
C39-C41	1.530(5)	C43-C44	1.489(5)
C44-C45	1.380(5)	C44-C49	1.389(5)
C45-C46	1.389(5)	C46-C47	1.368(6)
C47-C48	1.372(6)	C48-C49	1.390(6)
C50-C53	1.517(5)	C50-C52	1.522(5)
C50-C51	1.536(5)	C54-C55	1.520(5)
C54-C57	1.527(5)	C54-C56	1.520(5)

Table S12. Bond angles (°) for **7**.

N4-Si1-N3	69.10(12)	N4-Si1-S3	106.89(10)
N3-Si1-S3	83.21(9)	N4-Si1-Si2	114.99(10)
N3-Si1-Si2	110.23(10)	S3-Si1-Si2	138.11(5)
N4-Si1-S2	103.51(10)	N3-Si1-S2	169.20(10)
S3-Si1-S2	91.80(5)	Si2-Si1-S2	79.83(4)
N6-Si2-N5	69.20(12)	N6-Si2-Si1	106.20(10)
N5-Si2-Si1	111.99(10)	C2-S2-Si1	96.69(12)
C3-S3-Si1	101.23(12)	C1-N1-C2	109.9(3)
C1-N1-C16	122.9(3)	C2-N1-C16	126.4(3)
C1-N2-C3	109.7(3)	C1-N2-C4	126.3(3)
C3-N2-C4	123.6(3)	C28-N3-C35	130.3(3)
C28-N3-Si1	89.5(2)	C35-N3-Si1	140.0(2)
C28-N4-C39	130.4(3)	C28-N4-Si1	93.6(2)
C39-N4-Si1	135.1(2)	C43-N5-C50	129.7(3)
C43-N5-Si2	91.4(2)	C50-N5-Si2	131.7(2)
C43-N6-C54	129.1(3)	C43-N6-Si2	91.6(2)
C54-N6-Si2	137.9(2)	N2-C1-N1	105.8(3)
N2-C1-S1	127.1(3)	N1-C1-S1	127.1(3)
C3-C2-N1	106.9(3)	C3-C2-S2	123.8(3)
N1-C2-S2	128.9(3)	C2-C3-N2	107.6(3)
C2-C3-S3	126.3(3)	N2-C3-S3	125.9(3)
C9-C4-N2	117.6(3)	C9-C4-C5	123.9(3)
N2-C4-C5	118.4(3)	C6-C5-C4	116.3(3)
C6-C5-C13	122.7(5)	C4-C5-C13	120.8(5)
C6-C5-C13'	122.6(14)	C4-C5-C13'	120.4(14)
C7-C6-C5	121.7(4)	C6-C7-C8	120.4(4)
C9-C8-C7	120.9(4)	C4-C9-C8	116.7(3)
C4-C9-C10	121.0(3)	C8-C9-C10	122.3(3)
C9-C10-C12	112.6(3)	C9-C10-C11	111.4(3)
C12-C10-C11	111.4(3)	C5-C13-C15	112.0(7)
C5-C13-C14	112.4(7)	C15-C13-C14	111.0(6)
C15'-C13'-C5	117(2)	C15'-C13'-C14'	110.9(19)
C5-C13'-C14'	104(2)	C21-C16-C17	122.6(3)
C21-C16-N1	119.0(3)	C17-C16-N1	118.4(3)
C18-C17-C16	117.6(4)	C18-C17-C25	120.7(4)
C16-C17-C25	121.8(3)	C19-C18-C17	121.0(4)
C18-C19-C20	120.8(4)	C19-C20-C21	120.7(4)
C16-C21-C20	117.4(3)	C16-C21-C22	122.0(3)
C20-C21-C22	120.6(3)	C21-C22-C23	110.3(3)
C21-C22-C24	112.1(3)	C23-C22-C24	111.6(3)
C17-C25-C27	112.6(4)	C17-C25-C26	109.8(4)
C27-C25-C26	112.3(4)	N3-C28-N4	107.7(3)
N3-C28-C29	125.0(3)	N4-C28-C29	127.2(3)

N3-C28-Si1	56.31(17)	N4-C28-Si1	51.41(16)
C29-C28-Si1	175.5(3)	C30-C29-C34	120.6(4)
C30-C29-C28	121.2(4)	C34-C29-C28	118.1(4)
C29-C30-C31	117.9(5)	C32-C31-C30	118.3(5)
C33-C32-C31	122.6(6)	C32-C33-C34	119.5(6)
C29-C34-C33	121.0(5)	N3-C35-C37	113.4(3)
N3-C35-C38	110.1(3)	C37-C35-C38	109.4(3)
N3-C35-C36	105.2(3)	C37-C35-C36	109.4(3)
C38-C35-C36	109.3(3)	N4-C39-C42	107.3(3)
N4-C39-C40	113.6(3)	C42-C39-C40	109.1(3)
N4-C39-C41	107.3(3)	C42-C39-C41	112.7(3)
C40-C39-C41	107.0(3)	N6-C43-N5	105.6(3)
N6-C43-C44	126.5(3)	N5-C43-C44	127.6(3)
N6-C43-Si2	53.60(17)	N5-C43-Si2	53.39(17)
C44-C43-Si2	164.8(3)	C45-C44-C49	119.8(4)
C45-C44-C43	119.6(3)	C49-C44-C43	120.7(3)
C44-C45-C46	120.2(4)	C47-C46-C45	119.7(4)
C46-C47-C48	120.9(4)	C47-C48-C49	119.9(4)
C44-C49-C48	119.6(4)	N5-C50-C53	112.4(3)
N5-C50-C52	110.5(3)	C53-C50-C52	110.0(3)
N5-C50-C51	105.3(3)	C53-C50-C51	109.4(3)
C52-C50-C51	109.1(3)	N6-C54-C55	111.3(3)
N6-C54-C57	111.4(3)	C55-C54-C57	110.2(3)
N6-C54-C56	106.0(3)	C55-C54-C56	109.1(3)
C57-C54-C56	108.7(3)		
